# Primordial germ cell-derived macrophages as a platform to study host–pathogen interactions

**DOI:** 10.3389/fcell.2026.1684495

**Published:** 2026-09-30

**Authors:** Sudeepta Kumar Panda, Nima Dehdilani, Guillermo Tellez, Ricardo Corona Torres, Lorna Taylor, Charlotte Cody, Samer Halabi, Zhiguang Wu, Rachel Hawken, Mark P. Stevens, Mike J. McGrew

**Affiliations:** 1 The Roslin Institute and Royal (Dick) School of Veterinary Studies, University of Edinburgh, Edinburgh, United Kingdom; 2 Gujarat Biotechnology University, Gandhinagar, Gujarat, India; 3 Division of Physiology and Climatology, ICAR-Indian Veterinary Research Institute (IVRI), Izatnagar, India; 4 Cobb-Europe, Colchester, United Kingdom

**Keywords:** chicken, CRISPR/Cas9, MyD88, primordial germ line-derived macrophages, primordial germ cells

## Abstract

**Introduction:**

Chickens play a crucial role in global food security but are susceptible to a plethora of avian and zoonotic pathogens. Understanding the contribution of pathogen and host factors during infection is important for the design of strategies to control avian diseases, but it is hampered by the lack of reliable and varied avian cell lines for *in vitro* studies. Macrophages are key components of the innate immune system. They serve as a first line of defense against many infectious agents and help to orchestrate adaptive immune responses. Primary macrophages derived from chickens are limited in number, short-lived in culture, and difficult to genetically manipulate.

**Methods:**

Here, we established a protocol where chicken primordial germ cells are differentiated into macrophage-like cells, which provide a continuous *in vitro* supply of functionally active macrophages.

**Results:**

Chicken primordial germ cell-derived macrophages (PGCdMs) had similar molecular, transcriptional, and functional characteristics to chicken bone-marrow-derived macrophages (BMDMs). PGCdMs phagocytosed and killed *Salmonella enterica* serovar Typhimurium, a zoonotic pathogen commonly transmitted to humans via contaminated poultry products. Moreover, gene-edited PGCdMs were produced by applying CRISPR/Cas9 to the parental PGCs. The knockout of the major signaling molecule MyD88 in PGCdMs revealed that PGCdMs underwent rapid apoptosis after stimulation with lipopolysaccharide.

**Conclusions:**

Taken together, chicken PGCdMs will be a valuable cellular resource for studying mechanisms underlying resistance to avian and zoonotic diseases.

## Introduction

According to the Food and Agriculture Organization, an estimated 75 M broiler chickens were reared for meat in 2022, and laying hens produced 1.6 Tn eggs. However, diverse pathogens threaten avian health and welfare, causing economic burdens for farmers and consumers. Moreover, zoonotic pathogens transmitted via contaminated meat or eggs exert significant societal costs. Understanding the roles of pathogen and host factors in infections is important for designing strategies to control avian and zoonotic diseases.

Macrophages are central to innate immunity, regulating the adaptive immune response and fine-tuning body homeostasis (Hume, 2015). Macrophages act as a first line of defense against invading pathogens, marshalling immediate responses triggered by pathogen-associated molecular patterns (PAMPs), and maintaining surveillance throughout the body. Broadly, macrophages have three essential functions: phagocytosis, antigen presentation, and immunomodulation. In addition, macrophages play crucial roles in many biological processes, including generating reactive oxygen species, iron homeostasis, repair of tissue injury, and multiple metabolic functions (Chen et al., 2023). Macrophages detect pathogens through dedicated receptors for PAMPs. Extracellular invaders are phagocytosed, and the activation of downstream signaling pathways typically leads to the killing of internalized pathogens. However, some pathogens have evolved to evade killing by macrophages, such as infectious bursal disease viruses and *Salmonella* (Khatri and Sharma, 2007; He et al., 2018). Differences in the properties of macrophages may underlie the variation in heritable resistance or resilience of poultry to pathogens (Gul et al., 2022). Primary macrophages of chicken are produced by *in vitro* differentiation of the peripheral blood or bone marrow, providing a tool for studying host–pathogen interactions (Peng et al., 2020). However, studying this in primary macrophages is challenging owing to their relatively low numbers *in vivo* and their short lifespan in culture. Two immortalized chicken macrophage cell lines, HD11 and MQ-NCSU, have been produced by virus infection (Qureshi, 1998), and they are widely used to avoid these limitations. On the contrary, these cell lines do not faithfully reflect all aspects of the macrophage phenotype and are not readily amenable to precise genetic modification.

Here, we investigated the feasibility of differentiating macrophages from primordial germ cells (PGCs), which are lineage-restricted germ cells that can be isolated and cultured indefinitely from many chicken breeds. PGCs can be genetically modified *in vitro* and subsequently used to generate genetically modified chicken lines. As PGCs express the pluripotency factors *OCT4* (chicken *POU5f1*), *NANOG*, *SOX3*, and *PRDM14*, they spontaneously undergo differentiation into pluripotent cells called embryonic germ (EG) cells. In suspension culture, chicken EG cells formed embryoid bodies (EBs) and *in vitro* differentiated into ectoderm, mesoderm, and endoderm lineages. *In vivo*, injection of chicken EG cells contributed to chimeric chickens having various differentiated tissues, including the gonads (Park and Han, 2000). Chicken PGCs have been differentiated into fibroblast-like cells amenable to infection with avian and human influenza viruses (Idoko-Akoh et al., 2023; Long et al., 2019). Macrophages have previously been generated from pluripotent stem cells in mice, humans, and pigs, which proliferate finitely in culture and produce a limited number of cells (Ackermann et al., 2018; Meek et al., 2022; Takata et al., 2017). In chickens, chicken embryonic germ cells and blastodermal cells have been used to generate EBs (Park and Han, 2000; Wu et al., 2016; Xiong et al., 2015; Chen et al., 2023), but they have not yet been differentiated into myeloid cells, particularly macrophages.

Here, we demonstrate for the first time that PGCs cultured from transgenic chicken lines and two breeds of chicken can be differentiated into macrophage-like cells (PGCdMs). We show that chicken PGCdMs proliferate robustly and exhibit key morphological, transcriptional, immunological, and functional characteristics closely similar to chicken bone-marrow-derived macrophages (BMDMs). In addition, PGCdMs phagocytosed and controlled *Salmonella* and responded to bacterial lipopolysaccharide (LPS) by releasing pro-inflammatory reactive oxygen species and anti-inflammatory interleukin (IL)-10 secretion. Furthermore, PGCdMs were genetically ablated for *MyD88*, a crucial gene in the Toll-like receptor (TLR)4 pathway, and differed in their subsequent response to extracellular LPS stimulation.

## Results

### Directed differentiation of chicken PGCs into chicken macrophage-like cells

Chicken PGCs proliferate in single-cell suspension in a defined low-calcium serum-free medium containing fibroblast growth factor (FGF), activin, and ovotransferrin (FAOT) (Whyte et al., 2015). We adapted a protocol developed to produce macrophages from induced pluripotent stem cells and embryonic stem cells of mice, humans, and pigs (Takata et al., 2017; Ackermann et al., 2018; Gutbier et al., 2020; Meek et al., 2022) to differentiate chicken PGCs into chicken macrophages. In phase 1, PGC cultures were cultured in FAOT at normal physiological concentrations of CaCl_2_ (1.8 mM) to initiate clumping of the PGCs and formation of EBs and the induction of mesoderm (Figure 1A). In phase 2, the EBs were adhered to a culture plate coated with fibronectin in a medium containing fetal bovine serum (FBS), chicken stem cell factor (SCF), and chicken colony stimulation factor 1 (CSF1) to initiate hematopoietic precursor cell induction. In phase 3, SCF was replaced with chicken IL-3 to expand hematopoietic progenitors and initiate macrophage induction. IL-3 was removed after 48 h, and the culture was continued with CSF1 to generate suspension-naïve macrophage-like cells. In phase 4, the naïve suspension macrophages were expanded in culture with CSF1 on non-fibronectin-coated culture plates over several weeks until they terminally differentiated into adherent macrophage-like cells (PGCdMs).

**FIGURE 1:**
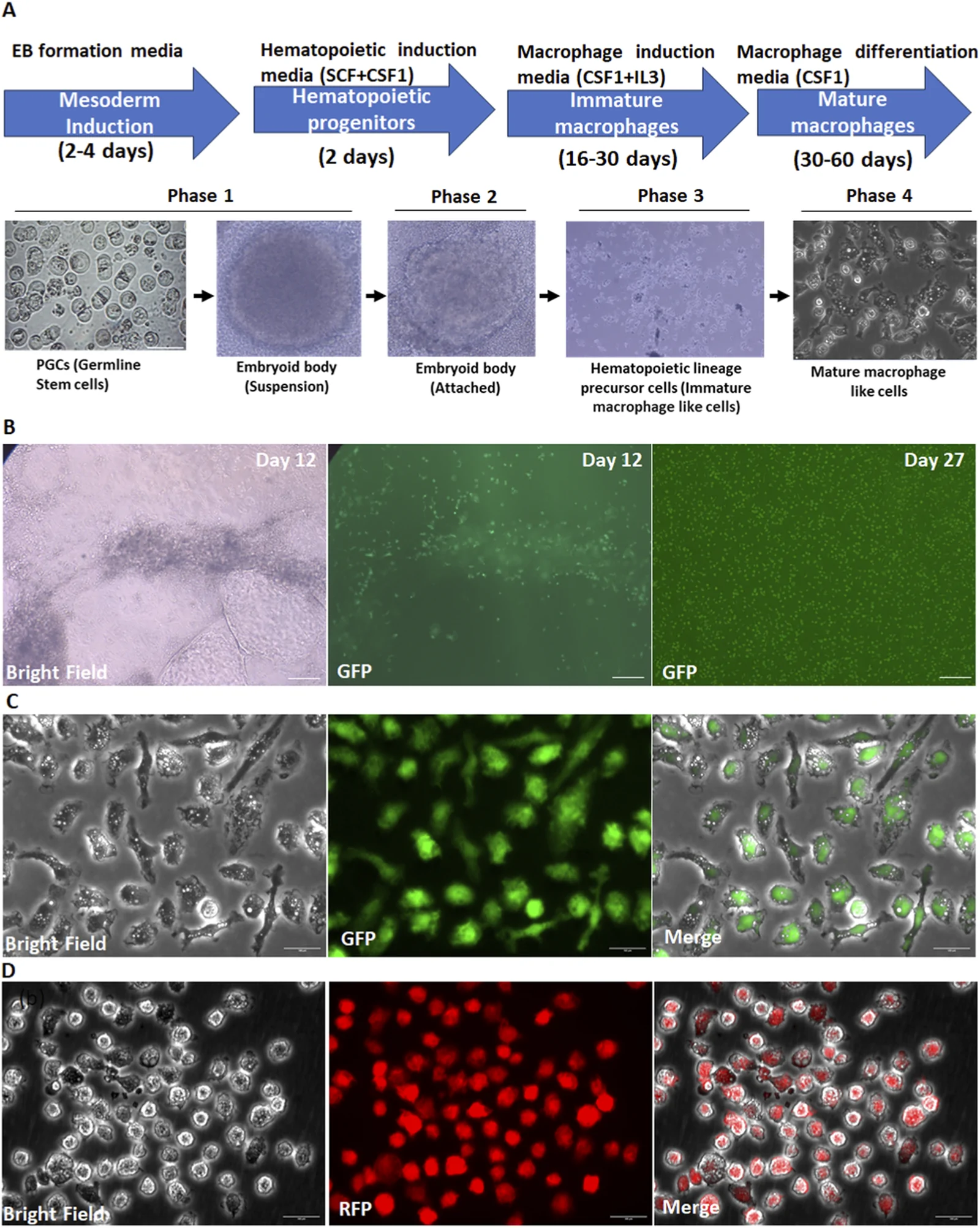
Differentiation of PGCs to macrophages. **(A)** Schematic of the four-phase PGCdM differentiation protocol. PGCs were first converted into embryoid bodies in normal CaCl_2_ medium to initiate mesoderm formation in phase 1. The culture medium was changed to hematopoietic induction media consisting of FBS, chicken stem-cell factor (SCF), and chicken colony-stimulating factor (CSF1) in phase 2. Phase 3 used the macrophage induction medium consisting of FBS, CSF1, and IL-3. Phase 4 used the macrophage differentiation medium consisting of FBS and CSF1. **(B)** Appearance of PGCdMs from *CSF1R*-GFP (MAC-Green) PGCs during phase-3 (day 12) and phase 4 (day 27). Scale bar, 100 μM. n = 3; data are representative of at least two independent experiments. **(C)** Morphological characteristics of PGCdMs (day 51) from MAC-Green PGCs. Scale bar, 50 μM. n = 3; data are representative of at least two independent experiments. **(D)** Morphological characteristics of PGCdMs (day 45) from MAC-Red PGCs. Scale bar, 50 μM. n = 3; data are representative of at least two independent experiments.

We developed and tested this protocol using a PGC line isolated from a *CSF1R*-eGFP transgenic chicken line (MAC-Green). The MAC-Green chicken line contains a green fluorescent reporter under the control of elements of the CSF1R promoter and enhancer that is active in myeloid cells at both embryonic and adult developmental stages (Balic et al., 2014; Garceau et al., 2015). The CSF1R promoter region is highly conserved in many bird species and contains binding sites of transcription factors AP1 and PU.1, which are both vital to activate hematopoietic lineages and initiate myelopoiesis (Raghav and Gangenahalli, 2018). MAC-Green PGCs were cultured in FAOT media with 1.8 mM CaCl_2_ for 2 days and formed EBs (phase 1) (Figure 1A; Supplementary Figure S1A). The EBs were transferred to a fibronectin-coated well and adhered to the well over 2 days (phase 2) (Supplementary Figure S1B). During phase 3, GFP^+^ cells were visible at approximately day 7–10 and appeared to emerge from a fibroblast-like cell population from larger fluorescent patches on the surface of EBs (Figure 1B; Supplementary Figure S1C). This GFP^+^ population had accumulated vacuoles and short processes (Supplementary Figure S1D). We observed a rapid proliferation of a single small GFP^+^ cluster on the plate to a larger fluorescent patch over 10 days after observing the first GFP^+^ cells (Supplementary Figure S2). At the same time, a few floating GFP^+^ cells were visible, emerging from the larger fluorescent patch, indicating some dividing cells had reduced adherence to the cell matrix.

We applied two methods of harvesting the GFP^+^ population and further passaged these cells. In the first method, once the GFP^+^ adherent patches had spread and reached confluency at approximately 3–5 weeks of differentiation, these adherent cells were detached by gentle pipetting and transferred to a new non-fibronectin-coated culture plate (phase 4), where they attached and exhibited macrophage morphology and continued expression of the GFP reporter (Supplementary Figure S3). In the second method, we alternatively harvested only these few floating GFP^+^’ from the original EB culture plates at approximately 10–14 days of differentiation, without disturbing the adherent GFP^+^ patches, every 3 to 5 days, and we continued the collection for approximately 4–5 weeks, until the collection culture plate surface was covered with GFP^+^ cells. Using both methods, a population of PGCdMs was recovered with minimal contamination of fibroblasts or other cell types (see Figure 2A). However, isolating floating cells generated a homogeneous population, with no fibroblast contamination, quicker than when the adherent GFP^+^ cells were first transferred to a new well. In phase 4, the harvested GFP^+^ cells continuously proliferated and were firmly attached to the culture plate in CSF1-containing media and produced adherent GFP^+^ macrophages. Once the adherent ‘macrophage-like cells’ were confluent, trypsin treatment was used to passage them in a 1:3 ratio to a new culture plate, and aliquots were frozen for subsequent use. We applied this isolation protocol to PGCs derived from an independent *CSF1R*-mApple transgenic reporter chicken line (MAC-Red) and obtained similar results (Balic et al., 2014) (Figure 1D).

**FIGURE 2:**
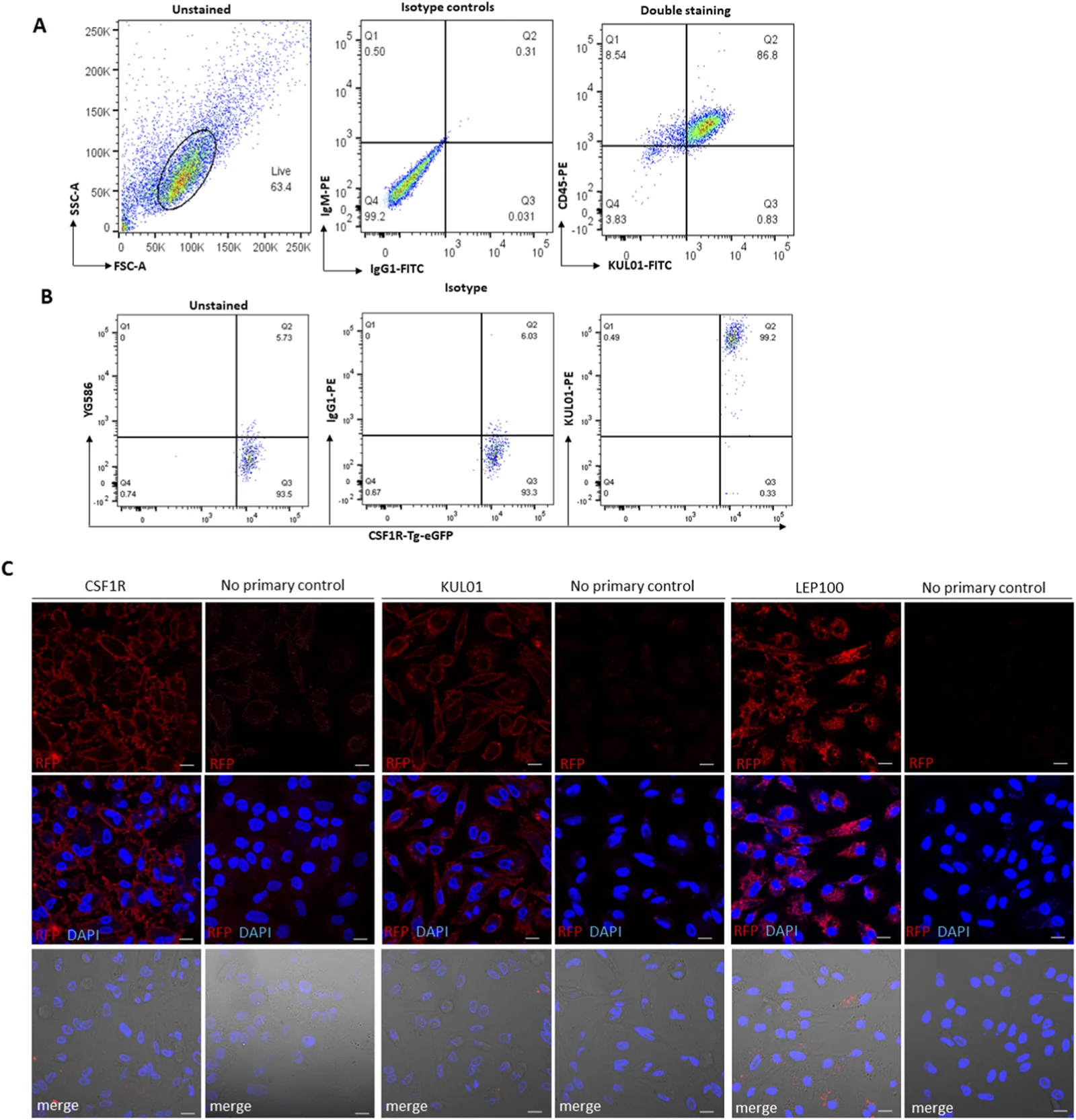
CD45, CSF1R, KUL01, and LEP100 are expressed on cultured PGCdMs. **(A)** Flow cytometry analysis of FR2M non-fluorescent PGCdMs immunostained with CD45-PE and KUL01 (MRC1L-B)-FITC, along with the isotype control. The CD45-PE was on the Y-axis, and KUL01-FITC was on the X-axis. **(B)** Flow cytometry analysis of MAC-Green GFP^+^ PGCdMs immunostained with KUL01-PE. KUL01-PE was on the Y-axis, and *CSF1R*-tg-eGFP was on the X-axis. **(C)** Confocal imaging of FR2M non-fluorescent PGCdMs immunostained with anti-*CSF1R*, KUL01, and LEP100 (Lamp1) antibodies. DAPI, nuclear stain. Merge, fluorescence and brightfield. Scale bars: 10 µM.

We next tested two variations of this method for the initial formation of EBs (Supplementary Figure S4). We cultured PGCs directly on a fibronectin-coated plate in 0.15 mM calcium chloride FAOT (method 2) or 1.8 mM calcium chloride FAOT media (method 3). This was to test the differentiation culture capabilities of PGCs to form EBs to generate putative macrophage-like cells. We compared derivations of PGCdMs using these three variations (see Materials and Methods). Data revealed that method 3 was more efficient in generating PGCdMs at a rate of 53% compared to method 1 (31%) and method 2 (25%) (Table 1). The overall success of generating PGCdMs from PGCs over the three methods was 33% (Table 1). Using all three methods, we succeeded in generating 39% transgenic PGCdM and 28% non-transgenic PGCdM lines from both broiler and layer chicken PGCs (Table 2). In conclusion, method 3 was the best method to generate PGCdMs from PGCs directly on a fibronectin-coated plate having 1.8 mM CaCl_2_ FAOT (Supplementary Table S1).

**TABLE 1:** Detailed number of derivations and the success rate from the three methods.

Method	Number of wells (n)	Successful derivations (n)	% Success
Method 1	45	14	31.1
Method 2	68	17	25
Method 3	32	17	53.1
Overall	145	48	33.1

**TABLE 2:** Number of cultures of transgenic and non-transgenic PGCs that lead to the generation of PGCdMs.

PGC lines	Number of wells (n)	Successful derivations (%)	Sex (male + female)
Transgenic reporter chicken lines	70	27 (38.57%)	14 M + 13 F
Non-transgenic chicken lines	75	21 (28%)	13 M + 8 F

Subsequently, we measured the proliferation rate and cell numbers of the PGCdMs. Surprisingly, PGCdMs continuously proliferated from a 24-well plate to T175 flasks for over seven passages without any sign of slower growth or gross morphological changes. Notably, PGCdMs could be frozen and re-cultured with a doubling time from 30 to 79 h, depending upon the parental PGC line (Supplementary Figure S5). Overall, these data indicate that the chicken PGCdMs retain a self-renewal capacity of cells that is distinct from mice, porcine, and human *in vitro*-derived macrophages.

### PGCdMs express macrophage and lysosomal markers

We examined the expression of the myeloid lineage surface marker CD45, the chicken macrophage-specific cell surface markers Mannose receptor C1-like B (MRC1L-B) recognized by KUL01 antibody, CSF1R, and MHC II on PGCdMs by flow cytometric analysis (Figure 2; Supplementary Figure S6). Immunostaining of cultured PGCdMs with CD45 and KUL01 showed that PGCdMs were CD45^+ve^ and MRC1L-B^+ve^ (Figure 2A) (86.8% double positive). Immunostaining of MAC-Green PGCdMs with KUL01 showed that PGCdMs were *CSF1R-Tg-*eGFP^+ve^ and MRC1L-B^+ve^, as compared to the control and isotype (Figure 2B) (99.2% double positive). Immunostaining of MAC-Green PGCdMs with MHC II revealed that a 1:500 dilution of MHC II-stained PGCdMs showed 15% MHC II^+ve^ eGFP^+ve^, whereas a 1:100 dilution led to 47% MHC II^+ve^ *CSFR1-Tg*-eGFP^+ve^, indicating a low expression level of MHC II protein on the surface of PGCdMs (Supplementary Figure S6A). However, MHC II expression was not detected in PGCdMs by immunofluorescence (Supplementary Figure S6B).

To further confirm the expression of macrophage markers by PGCdMs, we performed immunohistochemistry of PGCdMs with CSF1R, KUL01, and lysosomal marker (Lamp1) antibodies. Immunocytochemistry followed by confocal imaging revealed that PGCdMs expressed these three markers (Figure 2C). DF1 fibroblasts were negative for CSF1R and KUL01 (Supplementary Figure S7).

### PGCdMs express macrophage signature genes

The expression of genes characteristic of pluripotent stem cells (*NANOG*), embryonic mesoderm [*Brachyury (TBXT)*], and germ cells (*DAZL*) was compared between primordial germ cells and PGCdMs (Supplementary Figure S8). The transcript of the pluripotent stem cell marker *NANOG* was highly reduced in PGCdMs, indicating the loss of pluripotency. Transcripts for both the mesodermal and germ cell markers, *TBXT* and *DAZL*, were also absent in PGCdMs. In contrast, the expression of specific immune-related genes, *IL-10* (Meunier et al., 2025) and *IL-1B* (Peng et al., 2020), were expressed, consistent with the formation of cells of the myeloid lineage (Supplementary Figure S9A-C). Expression of other immune-related genes, such as *MyD88, MHC II, BAX, TNF-α, IL-6, iNOS, and MDA5,* was approximately equal in PGCs and PGCdMs (Supplementary Figure S9A-C).

### The transcriptome of PGCdMs is distinct from that of PGCs and similar to that of bone-marrow-derived macrophages

To further characterize PGCdMs, we compared the transcriptome of PGCdMs derived from five different PGC lines (F7MAG, FR2M, M16MAG, F11MAA, and Cobb500) encompassing two breeds of chicken, with PGCs, DF1 cells (a chicken fibroblast cell line), and bone-marrow-derived chicken macrophages (BMDMs) (Idoko-Akoh et al., 2023; Borowska et al., 2022; Niu et al., 2017). A principal component analysis (PCA) revealed a distinct transcriptomic profile between PGCdMs, PGCs, and DF1 cells. PGCdMs cluster with BMDMs, showing that they have a broadly similar transcriptome (Figure 3A). The biological replicates in each category are shown to have a consistent Cook’s distance from each other, which indicates no outliers within each cell type (Supplementary Figure S10). The transcriptomic profile of the top 1,000 variable genes among these four cell types revealed that PGCdMs differed from the other cell types and clustered most closely with BMDMs (Figure 3B). Next, we identified 14,885 differentially expressed genes (DEGs) in PGCdMs relative to PGCs. As expected, the *DAZL*, *NANOG*, *POU5f3*, *DMRT1*, *CLDN1, SOX3*, *DMRTB1*, and *CBX2* core signature genes of germ cells were highly expressed in PGCs than in the three other cell types in the heat map, confirming their germline identity (Figure 3C). The fibroblast core signature genes *PDGFRA*, *VIM*, *COL1a1*, *LOXL1*, *COL5a1*, *TWIST2*, *PLEK2*, and *COLEC10* were highly expressed in the DF1 cell line and partially expressed in BMDMs. Notably, BMDMs and PGCdMs exhibited a distinct enrichment of committed macrophage core signature genes such as *TLR4*, *TLR7*, *CSF3R*, *TNFRSF21*, and *PSTPIP2* (Figure 3C). Moreover, RNA sequencing data of PGCdMs showed the expression (log2FC > 3) of *BLB2, DMA, CD74, CSTA (cathepsin A), CSTB (cathepsin B), CSTC (cathepsin C), CTSD (cathepsin D), CTSK (cathepsin K)*, CTSS (*cathepsin S),* and *CTSZ (cathepsin Z)*—the key genes of chicken MHC class II system (Supplementary Figure S6C) (Parker and Kaufman, 2017). The RNA expression data of the MHC II gene family in PGCdMs partially validates the MHC II antibody staining of PGCdMs, though they were negative for MHC II via immunostaining (Supplementary Figure S6B). Overall, these findings confirm that PGCdMs have transcriptional characteristics of the monocyte-macrophage lineage.

**FIGURE 3:**
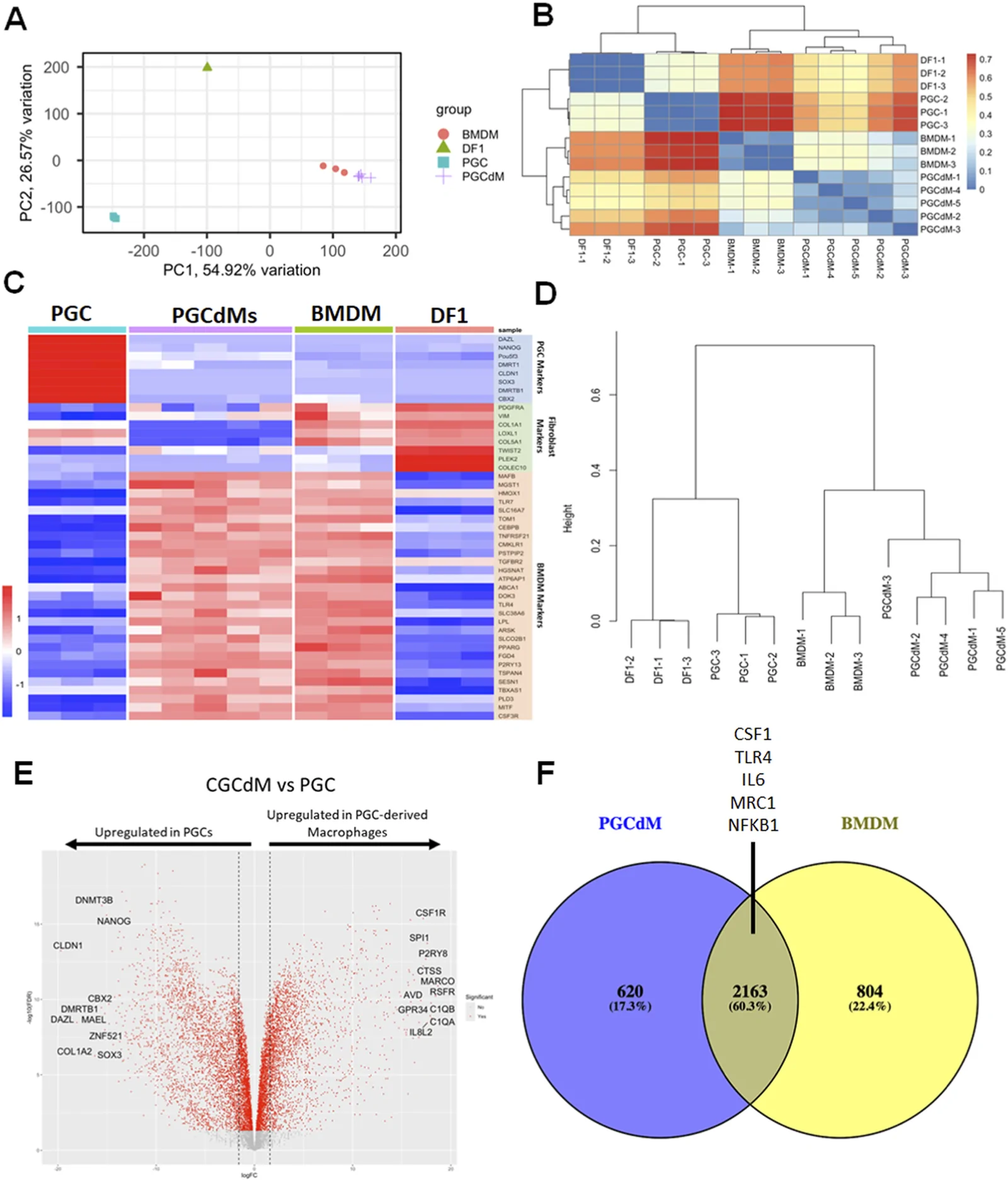
Transcriptome comparison between PGCdMs and other cell types. **(A)** PCA plot depicting the clustering of PGC-derived macrophages with bone-marrow-derived macrophages and dendritic cells differs from DF-1 and PGCs, based on the top 1,000 variable genes. n = 3 cell lines per group. **(B)** Pearson correlation of PGCdMs in comparison to other cell types. n = 3 cell lines per group. **(C)** Heat map showing the sample distances are based on 100 variable genes in PGCdMs, in contrast to that in other cell types. n = 3 cell lines per group. **(D)** Dendrogram showing the relatedness of or dissimilarities between PGCdMs with other cell types. n = 3 cell lines per group. **(E)** Volcano plot showing differentially expressed genes between PGCs and PGC-derived macrophages based on an average expression value higher than 500. Significant DEGs are shown as red dots (|log2FC| < 3, and FDR 0.05). **(F)** A Venn diagram showing the number of genes expressed in PGC-derived macrophages and bone marrow-derived macrophages. Genes with an average normalized read count higher than 500 were expressed in a particular cell type.

We calculated the distance between the different samples using only DEGs by constructing dendrograms. Sample- and gene-based hierarchical cluster analyses revealed similarities among the samples in the four different groups (Figure 3D). Consistent with the findings above, differential gene expression (DEG) analysis between PGCdMs and PGCs revealed a distinct enrichment of macrophage-specific core signature genes such as *CSF1R*, *SPI1*, *MARCO*, and *IL8L2* in PGCdMs. Conversely, PGCs exhibited robust expression of known PGC markers, including *DAZL, NANOG,* and *SOX3*, as expected (Figure 3E; Supplementary Figure S11). Notably, PGCdMs and BMDMs shared 60.3% of DEGs, showing the distinct enrichment of committed hematopoietic lineages, such as *CSF1*, *TLR4*, *IL-6*, *MRC1*, and *NFKB*, indicating further commitment toward a monocyte-macrophage lineage (Figure 3F). KEGG pathway enrichment analysis of DEGs between PGCdMs vs. PGCs (log2FC > 1.5) and BMDM vs. PGCs (log2FC > 1.5) identified 22 signaling pathways that were significantly enriched in PGCdMs and BMDMs (Supplementary Figure S12A). These included the most predominant pathways, such as the TLR, RIG-1, JAK-STAT, NF-kappa B, TNF, NOD-like receptor, Fc gamma R-mediated, and the chemokine signaling pathway, known to regulate phagocytosis, C-type lectins, and efferocytosis receptors (Supplementary Figure S12B). A GO term enrichment analysis of DEGs between PGCdMs and PGCs (log2FC > 4) revealed enrichment of 34 terms, whereas GO term enrichment analysis of DEGs between BMDMs and PGCs (log2FC > 4) revealed enrichment of 27 terms (Supplementary Figure S13). However, both PGCdMs and BMDMs have significant enrichment of GO terms associated with immune response, antigen processing, presentation, apoptosis, phagocytosis, neutrophil chemotaxis, and various immune cell activation and suppression. Subsequently, we compared the relative gene expression for the top macrophage signature genes, revealed in each KEGG pathway, in between four sets of cell types (Supplementary Figure S12B). Notably, these distinctive core signature genes, specific to various macrophage signaling pathways such as *TLR, NFKB, JAK-STAT, MAPK, TNF,* chemokine, FcGR, phagocytosis, *NOD1, mTOR, PAPAR,* and *PI3K-AKT*, showed a similar gene expression pattern in PGCdMs and BMDMs and were not expressed in PGCs and DF1 cells (Supplementary Figure S12B). These findings indicate that PGCdMs acquire a cellular identity closely resembling bone-marrow-derived macrophages. The DEGs between PGCdMs vs. PGCs, PGCdMs vs. BMDMs, and PGCdMs vs. DF1 are shown in Supplementary Data Set 1–3.

### PGCdMs phagocytose yeast particles

We next analyzed the extent to which PGCdMs are capable of phagocytosis. PGCdMs were incubated with fluorophore-labeled yeast particles (pHrodo beads). The chicken DF1 fibroblast cell line was used as a negative control. pHrodo beads fluoresce at low pH in the acidic cell environment of phagosomes. At 4, 8, and 24 h of incubation, red fluorescence and % pHrodo positive cells were significantly increased in PGCdMs compared to that in the control (Figure 4; Supplementary Figure S14). Control DF1 cells had low levels of fluorescence over the same time course in comparison to that in PGCdMs (Supplementary Figure S15). Live cell imaging captures the dynamics of the phagocytosis process (Supplementary Video S1).

**FIGURE 4:**
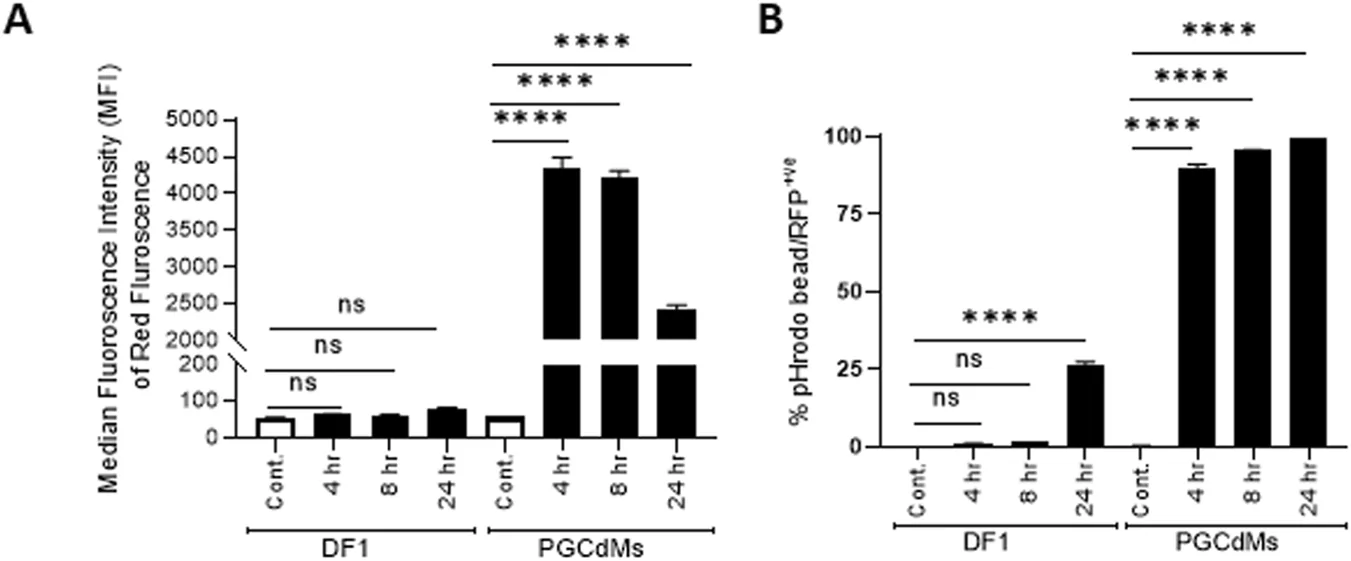
Phagocytosis by PGCdMs. PGCdMs and DF1 cells were incubated with pHrodo yeast beads and observed for red fluorescence as an indicator of phagocytosis activity. **(A)** Comparing the median fluorescence intensity of pHrodo bead uptake in DF1 and PGCdMs in 0, 4, 8, and 24 h ns = non-significant; ****P = <0.0001. Tukey’s ordinary one-way ANOVA test with no matching/pairing was applied. **(B)** Comparing the percentage of pHrodo bead uptake in DF1 and PGCdMs at 0, 4, 8, and 24 h ns = non-significant; ****P = <0.0001. Tukey’s ordinary one-way ANOVA test with no matching/pairing was applied.

### Production of reactive oxygen species increases in LPS-stimulated PGCdMs

To determine whether PGCdMs recognize pathogen-associated molecular patterns and initiate relevant downstream signaling pathways to elicit further immune responses, we exposed PGCdMs to bacterial LPS, which is recognized by TLR4, and investigated whether they released pro-inflammatory and anti-inflammatory mediators. Macrophages produce nitric oxide (NO) after stimulation with LPS, which contributes to the killing of internalized pathogens including parasites, fungi, and bacteria (Wu et al., 2016). NO production by the PGCdMs and BMDMs was analyzed after LPS stimulation (500 ng/ml) compared to that in unstimulated cells at 4, 8, and 24 h. At 4 h, NO was not detected in control BMDMs with or without LPS. There was significant NO production at 8 h of LPS stimulation in BMDMs, which significantly increased at 24 h of LPS stimulation. Production of NO was not detected in PGCdMs with or without LPS for 4 and 8 h. However, after 24 h of LPS stimulation, PGCdMs produced significantly higher levels of NO, as was observed with LPS-stimulated BMDMs (Figure 5A).

**FIGURE 5:**
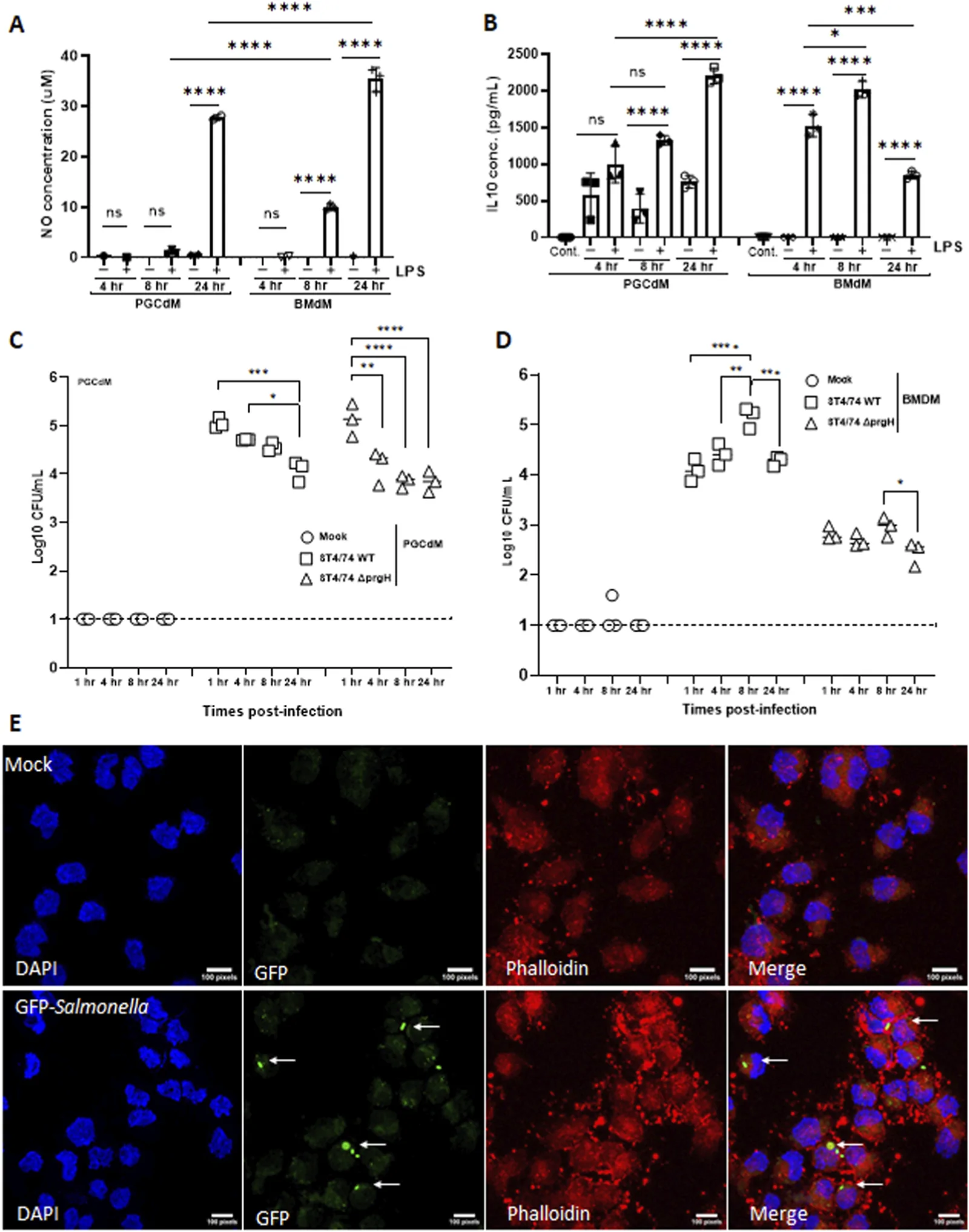
PGCdMs respond to LPS and phagocytose and control *Salmonella*. **(A)** Production of reactive oxygen species by LPS-stimulated PGCdMs and BMDMs. The PGCdMs and BMDMs were challenged with LPS for periods up to 24 h. LPS-stimulated supernatants were examined for ROS using the Griess assay. n = 3; data are representative of at least two independent experiments. In PGCdMs: unstimulated vs. LPS stimulated at 24 h, ****P = <0.0001; ns = non-significant. In BMDMs: unstimulated vs. LPS stimulated at 8 h, ****P = <0.0001; unstimulated vs. LPS stimulated at 24 h, ****P = <0.0001; ns = non-significant. Tukey’s one-way ANOVA test was applied. **(B)** IL-10 secretion from PGCdMs. The PGCdMs and BMDMs were challenged with LPS for up to 24 h. LPS-stimulated supernatants were examined for IL-10 secretion by IL-10 sandwich ELISA. n = 3; data are representative of at least two independent experiments. ****P = <0.0001; ***P = 0.0004; *P = 0.0147; ns = non-significant. Tukey’s one-way ANOVA test was applied. **(C)** *Salmonella enterica* serovar Typhimurium infection of PGCdMs. PGCdMs were infected with GFP expressing *Salmonella* strains ST4/74 wild-type (left) or ST4/74 D*prgH* mutant (right). After gentamicin treatment, the PGCdMs were lysed 1, 4, 8, or 24 h after the addition of bacteria to quantify intracellular gentamicin-protected bacteria. n = 3; data are representative of at least two independent experiments. *P = 0.0348; **P = 0.0010, ***P = 0.0008; *****P = <0.0001. Tukey’s one-way ANOVA test was applied. **(D)** *Salmonella* infection of BMDMs. Net replication of ST4/74 wild-type (left) or ST4/74 D*prgH* was assayed in BMDM as above for PGCdMs. n = 3; data are representative of at least two independent experiments. **P = 0.0016, ***P = 0.0002; *****P = <0.0001; ns = non-significant. Tukey’s one-way ANOVA multiple comparison tests were used to check the statistical difference between various conditions. Scale bar, 10 µM. **(E)** Confocal Z-stacked projected images of PGCdMs infected with *Salmonella* ST4/74 compared to mock. After 1 h of infection, infected PGCdMs were gentamicin-treated, fixed and permeabilized, and stained for DNA DAPI (blue), F-actin with phalloidin (red), and *Salmonella* was visualized owing to the expression of GFP. The upper panels show mock-infected and the lower panels show *Salmonella*-infected PGCdMs. The white arrow indicates the phagocytosed GFP expressing *Salmonella* in PGCdMs. Scale bar, 10 μM. n = 3; data are representative of at least two independent experiments.

### IL-10 production increases over time in LPS-stimulated PGCdMs

Interleukin-10 is an anti-inflammatory cytokine that controls the inflammatory responses during infection with viruses, bacteria, fungi, protozoa, and helminths and plays a central role in homeostasis (Couper et al., 2008). We determined whether LPS stimulation (500 ng/ml) of PGCdMs induced IL-10. PGCdMs produced higher levels of IL-10 at all the time intervals analyzed in the absence of LPS compared to BMDs (Figure 5B). At 8 h, LPS-stimulated PGCdMs produced significantly higher IL-10 compared to that in unstimulated cells, similar to BMDMs’ response to LPS. At 24 h of LPS stimulation, PGCdMs produced significantly higher IL-10 than that produced at 8 and 4 h. The LPS-stimulated IL-10 secretion for BMDMs showed a similar pattern to that previously described (Wu et al., 2016).

### Phagocytosis and control of *Salmonella* within the PGCdMs

To evaluate the suitability of PGCdMs as a host–pathogen interaction model equivalent to the BMDM model, we challenged PGCdMs with a *Salmonella enterica* serovar Typhimurium strain proven to efficiently colonize the chicken gastrointestinal tract and cause systemic disease in neonatal chicks. PGCdMs and BMDMs were challenged with a *S.* Typhimurium ST4/74 *nal* ^*R*^ (WT STm) and a non-invasive mutant lacking the function of type 3 secretion system 1, which induces phagocytosis of the bacterium by epithelial cells (Δ*prgH* STm). Both the strains were engineered to express enhanced green fluorescent protein (EGFP). After infection for 30 min, gentamicin was added to the culture medium to kill extracellular bacteria. Intracellular gentamicin-protected bacteria were then enumerated at 1, 4, 8, and 24 h after the bacteria were initially added. In PGCdMs, both the WT and Δ*prgH* mutants were efficiently phagocytosed at comparable levels, indicating that the uptake is not a consequence of phagocytosis triggered by the type 3 secretion system (Figure 5C). Over time, the number of viable intracellular bacteria of both strains declined, indicating that the PGCdMs have antimicrobial activity (Figure 5C). By comparison, in BMDMs, a subset of the bacteria appears to have been internalized as a consequence of T3SS-mediated phagocytosis, as the number of internalized bacteria was lower for the Δ*prgH* mutant than for the wild-type (Figure 5D). Moreover, the number of intracellular bacteria did not significantly decrease over time. Uptake of the bacterial strains by PGCdMs was confirmed by confocal microscopy (Figure 5E). However, the available image resolution does not allow intracellular *Salmonella* to be definitively distinguished from bacteria associated with the cell surface. Higher-resolution imaging and/or additional cell-membrane staining would be required to conclusively confirm intracellular localization.

### Genome editing of the TLR pathway gene *MyD88* in chicken PGCdMs

We asked whether genome-edited PGCs could be used to investigate macrophage gene function in PGCdMs. *MyD88* is a cytosolic adapter protein central to the response of pattern recognition receptors. To delete the *MyD88* gene from PGCdMs, PGCs isolated from the MAC-Green transgenic reporter chicken were transfected with a pair of guides designed to delete the *MyD88* coding regions by cutting 13 bp after the translational start site and 48 bp after the termination codon (Figure 6A). From the post-transfected heterogeneous gene-edited population, single-cell PGC clones were isolated by FACS sorting and screened for deletions by a genotyping PCR (Figure 6B). Out of 120 single-cell clones set for clonal dilution, seven clones survived, and 6/7 clones carried a deleted *MyD88* allele, of which three clones were homozygous for the deletion (Table 3). Sanger sequencing of the *MyD88* PCR products identified the guide sequence of the gRNA targeting exon1 immediately followed by the guide sequence of sgRNA targeting the 3’ UTR, confirming the 9.2-kb deletion fragment in the *MyD88* locus and revalidating the genotyping PCR data (Supplementary Figure S16). These three independent homozygous *MyD88* ^−/−^ clones were successfully differentiated into PGCdMs, as shown by their adherent morphology and expression of the MAC-Green (*CSF1R*-GFP) reporter (Figure 6C). TLR4 expression in KO PGCdMs did not change in comparison to wild-type PGCdMs (Supplementary Figure S17B).

**FIGURE 6:**
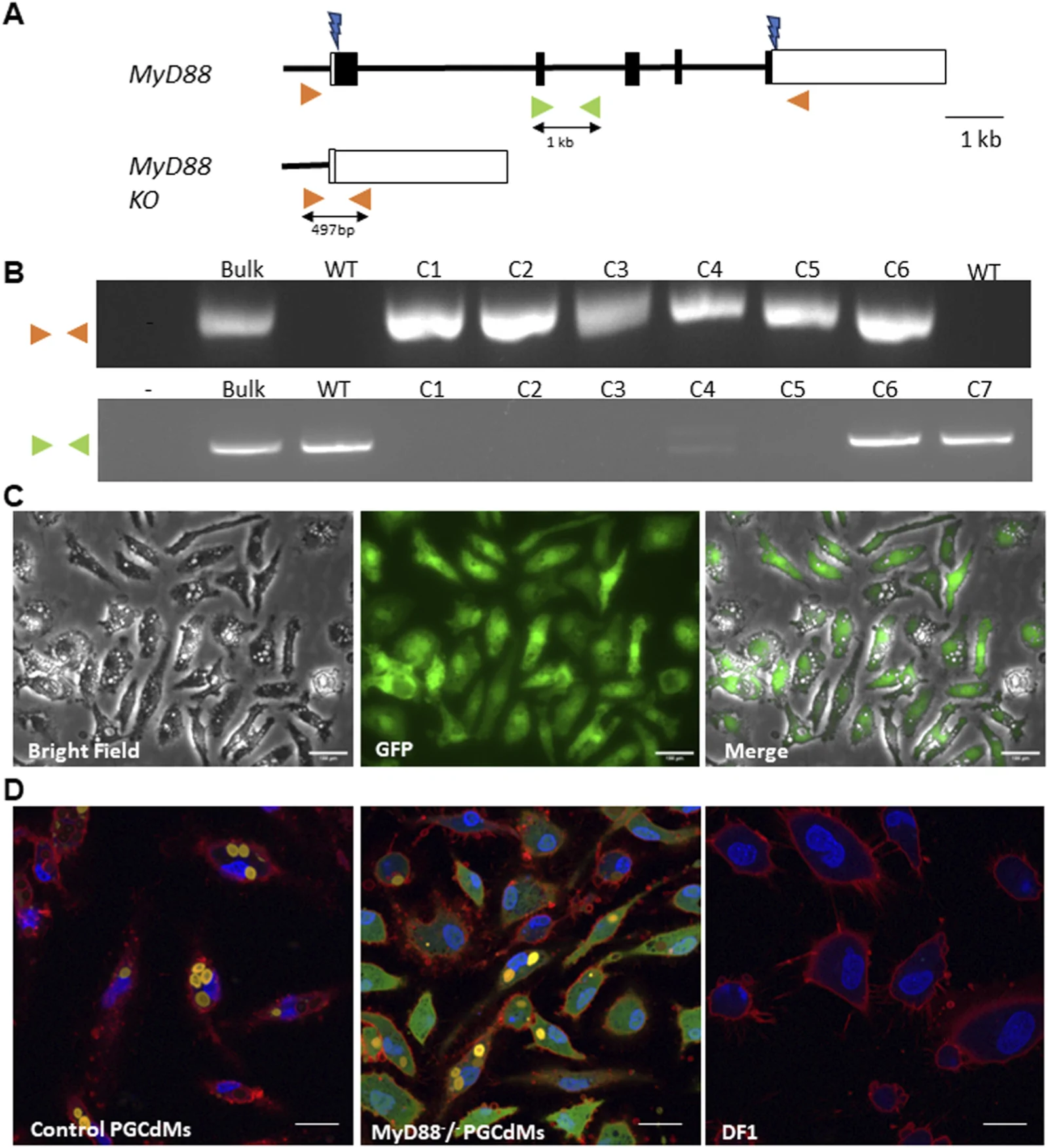
*MyD88* gene editing of chicken PGCdMs. **(A)** *MyD88* gene structure using a two-guide strategy to generate a deleted allele. The blue lightning bolts designate the Cas9 cleavage site 14-bp upstream of the translation start site and 45-bp upstream of the stop codon. Coding exons are shown in black boxes, 5′ and 3′ UTRs are shown as white boxes. Orange arrowheads show PCR primers used to detect the deleted allele. Green arrowheads show PCR primers used to detect wild-type allele. **(B)** Upper panel shows the PCR analysis that showed the expected *MyD88* knockout allele for single-cell clones using the orange arrowhead primers to detect the deleted allele. The lower panel shows the PCR using the green arrowhead primers to detect the wild-type allele. -, no DNA; +, parental PGC line. **(C)** GFP fluorescence of MAC-Green PGCdMs generated from a *MyD88* ^*−/−*^ PGC clone. Scale bar, 50 μM. n = 3; data are representative of at least two independent experiments. **(D)** Fluorescence confocal micrographs of wild-type and *MyD88* ^−/−^ PGCdMs incubated with pHrodo beads at 4 h of incubation. DAPI, nuclear stain. Plasma membrane stained with Cell Mask Deep Red. Scale bar, 10 µM. Data are representative of at least three independent experiments.

**TABLE 3:** *MyD88* editing efficiency.

No. of targeted PGCs plated for clonal growth	Surviving colonies	Genotype wild-type (+/+)	Genotype heterozygous (+/−)	Genotype homozygous(−/−)
120	7	1 (14%)	3 (42%)	3 (42%)

Furthermore, sequencing of the putative top-three off-target sites of sgRNA1 in *MyD88* ^−/−^ macrophages identified no deletions at these sites (Supplementary Figure S18). In addition, PCR for the plasmid revealed no integration of the targeting plasmid in the *MyD88* ^−/−^ macrophages (Supplementary Figure S19). Furthermore, RT-PCR and sequencing analysis revealed that the *MyD88* expression was ablated in *MyD88* KO PGCDMs compared to wild-type PGCdMs (Supplementary Figure S17C; Supplementary Figure S20).

#### *MyD88* ablation does not affect phagocytosis by PGCdMs

We investigated whether the absence of *MyD88* affects phagocytosis of fluorophore-labeled yeast particles by edited PGCdMs. Fluorescent microscopy revealed that most of the *MyD88* ^−/−^ macrophages exhibited red fluorescence by 4 h of incubation compared to control DF1 cells, indicating that *MyD88* KO macrophages had efficiently taken up the yeast beads (Figure 6D). Confocal imaging using z-sections and plasma membrane staining confirmed that the pHrodo beads were internal in both the wild-type and the *MyD88* ^−/−^ PGCdMs and did not adhere to the cell surface (Figure 6D; Supplementary Figure S21). A time-lapse video captures the dynamics of this process (Supplementary Video S2). This result was expected as the function of *MyD88* in phagocytosis is complemented by TLR3/TRIF activation to activate PI3K that triggers actin polymerization to initiate the phagocytosis in *MyD88* KO mice (Shin et al., 2009). Overall, these data support the previous finding that *MyD88* ablation in macrophages is not sufficient to prevent phagocytosis.

### *MyD88* ^*−/−*^ PGCdMs cells undergo rapid apoptosis upon LPS stimulation

To functionally investigate the role of *MyD88* in chicken PGCdMs in producing pro-inflammatory mediators of phagocytosis, both control and *MyD88* ^−/−^ PGCdMs were stimulated with 500 ng/mL LPS for 4 h. At the endpoint of stimulation, microscopic observation revealed that wild-type macrophages stimulated with LPS appeared to adopt a mixed macrophage/dendritic cell-like phenotype (Figure 7A). However, unexpectedly, *MyD88* ^−/−^ macrophages stimulated with LPS rapidly died (Figure 7B, Supplementary Video S3). A similar result has not been shown for mammalian macrophages and indicates that *MyD88* plays a distinct role in avian species.

**FIGURE 7:**
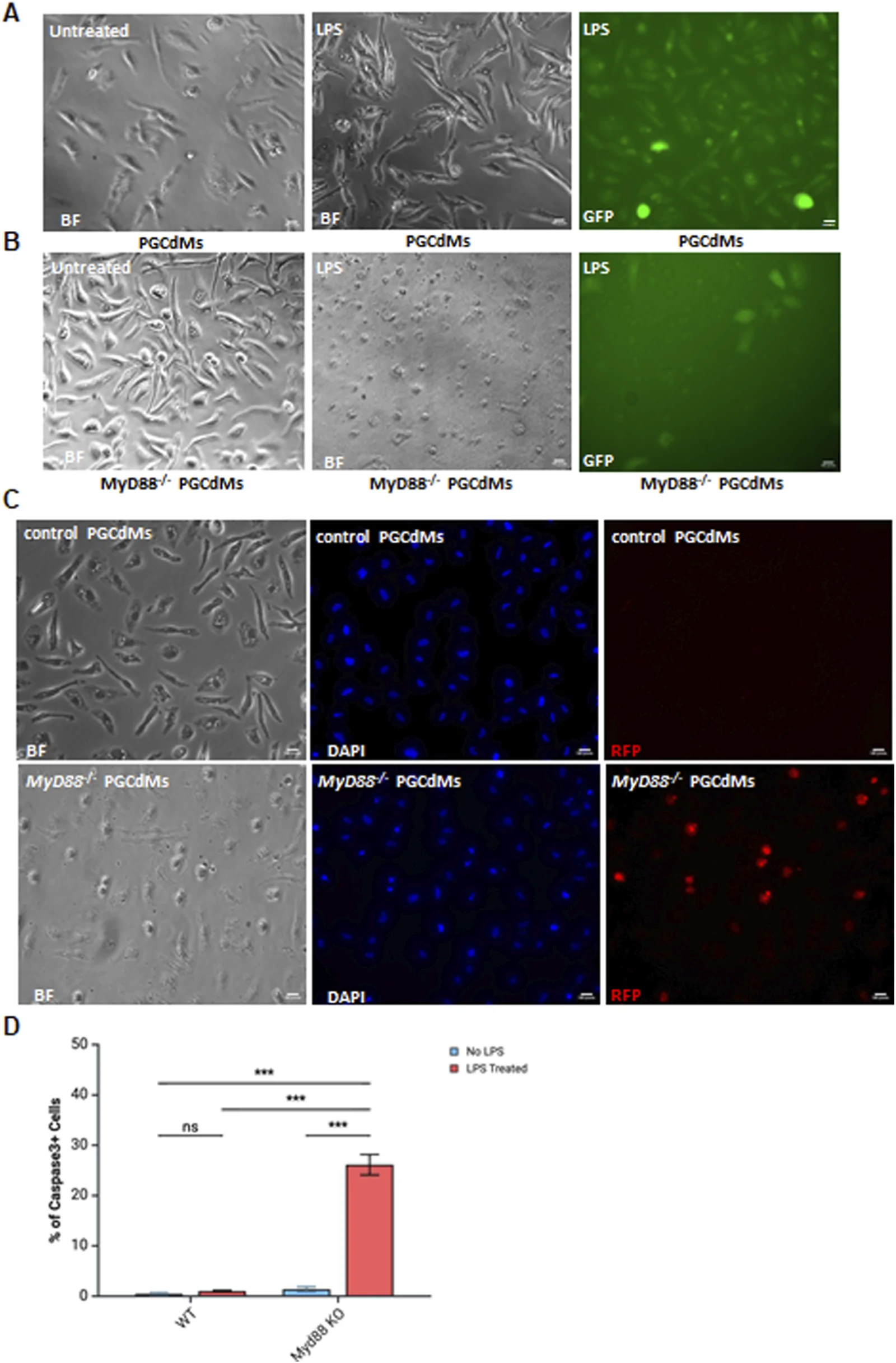
LPS induces apoptosis in *MyD88* ^*−/−*^ PGCdMs; **(A)** control and **(B)** *MyD88* ^−/−^ PGCdMs stimulated with 500 ng/mL LPS. **(C)** Control (upper panel) and *MyD88* ^−/−^ (lower panel) macrophages were stimulated with LPS 250 ng/mL for 1 h and immunostained for cleaved caspase-3. **(D)** Quantification of RFP^+^ cells for control and *MyD88* ^−/−^ PGCdMs stimulated with LPS. A–C, Scale bar, 10 µM.

To determine whether LPS stimulation in *MyD88* ^−/−^ PGCdMs led to caspase-3 cleavage, activation, and apoptosis via caspase-8, PGCdMs were stimulated with a lower concentration of LPS (250 ng/mL) for 1 h and immunostained with an antibody to cleaved caspase-3. A lower dose of LPS led to a few surviving *MyD88* ^−/−^ PGCdMs. Immunocytochemistry data revealed that LPS-stimulated *MyD88* ^−/−^ PGCdMs expressed cleaved caspase-3 compared to unstimulated *MyD88* ^−/−^ PGCdMs, which had no cleaved caspase-3 staining (Figure 7C). Quantification of the caspase-3 signal in the viable LPS-stimulated *MyD88* ^−/−^ PGCdMs indicated that 30% of these cells contained cleaved caspase-3 signal compared to wild-type PGCdMs (Figure 7D). Furthermore, staining for annexin-V, another marker of apoptosis, showed increase in the % of late apoptotic and dead/necrotic c *MyD88* ^−/−^ PGCdMs after treatment with LPS as compared to that in wild-type (Supplementary Figures S22 and S23).

## Discussion

Our four-step protocol for differentiating chicken PGCs to PGCdMs is adapted and modified from the methods devised for the differentiation of porcine, mouse, and human pluripotent stem cells to macrophages (Meek et al., 2022; Lopez-Yrigoyen et al., 2018). The differentiation of chicken PGCs in a 24-well plate typically produced attached EBs in 4 days after the start of differentiation and required another 7–30 days to begin to generate of *CSF1R*-GFP^+^ cells, after which they continuously produced PGCdMs for an additional 4 weeks. Simply directly adhering PGCs to each other in a coated plate (method 3) proved as efficient at generating PGCdMs as the transfer of EBs (Supplementary Table S1). In addition, the isolation of floating macrophage precursors eased isolation by avoiding trypsinization and dislodging of adherent cells from the well surface.

Detection of the *CSF1* receptor on PGCs-derived macrophages indicates that they are monocyte-macrophage-like cells (Figure 2). This is further evident from expression of the *CSF1R*-eGFP/RFP transgenes in PGCdMs derived from MAC reporter chicken lines. The *CSF1R* regulates macrophages’ proliferation, differentiation, and survival (Rojo et al., 2019). Though the function of *CSF1R* is conserved in all amniotes (Hume et al., 2017), the germline deletion of *CSF1R* in mice and rats significantly reduced most tissue macrophage populations, leading to defective phagocytosis, microglial deficiency, osteoporosis, reproductive defects, increased primitive hematopoietic progenitor cells, and depleted tissue-resident macrophages (Dai et al., 2002; Pridans et al., 2018).

Transcriptome analysis indicated that the gene expression in PGCdMs overlapped significantly with that of chicken BMDMs (Figure 3). This included the expression of core signature genes that identify tissue-resident macrophages (Gautier et al., 2012). In addition, PGCdMs expressed canonical and non-canonical inflammatory signaling pathways that are also found in tissue-resident macrophages (Supplementary Figure S12B). Moreover, KEGG and Gene Ontology pathway analysis of DEGs of PGCdMs identified the signaling pathways that are linked to innate immunity and a part of adaptive immunity (Supplementary Figures S12, S13).

Chicken PGCdMs also exhibited functional attributes of *ex vivo* and *in vivo* macrophages. PGCdMs were phagocytic and accumulated the intracellular pathogen *Salmonella* over time (Figure 5C). However, our data do not prove that this accumulation was intracellular. With our protocols, it will now be feasible for researchers to explore the use of PGCdMs as models for other avian and zoonotic pathogens and to determine if differential resistance of avian lines or breeds can be reproduced in PGCdMs derived from their PGCs.

Phagocytosis by macrophages is an important arm of innate immunity. *MyD88* signaling is vital to limit bacterial burden and prolong survival during infection. Upon binding ligands to TLRs, activated downstream signaling cascades signal through the *MyD88* adapter protein to induce the production of inflammatory cytokines in macrophages and dendritic cells. These include interferon-α, interferon-β, interleukin 12, tumor necrosis factor α, interleukin 6, and interleukin 1, which leads to the activation of innate immune cells and the initiation of adaptive immune responses (Hou et al., 2008). *MyD88* KO mice have increased susceptibility to bacterial and viral infection. For example, they are more susceptible to primary influenza virus infection and need to be raised in a specific pathogen-free environment (Seo et al., 2010). Defects in the toll-like receptor (TLR4) signaling pathway hamper phagocytosis (Blander and Medzhitov, 2004). In our study, the deletion of *MyD88* in macrophages did not affect their phagocytic activity of yeast particles (Figure 6D). These data are in line with the previous finding that showed that the absence of *MyD88* was complemented by TRIF activation via the convergence of the signaling pathway downstream of *MyD88* and TRIF that activates downstream PI3K, which triggers actin polymerization to initiate phagocytosis (Shin et al., 2009). In our study, LPS-mediated TLR4 signaling in *MyD88* ^−/−^ chicken PGCdMs unexpectedly triggered rapid apoptosis and activated caspase-3 (Figure 7, Supplementary Video S3). This contrasts with observations in *MyD88* KO mice in response to LPS challenge (Kawai et al., 1999) and may indicate species-specific differences in the activity of MyD88. Whether this system models tissue-resident vs. monocyte-derived macrophages needs to be studied further. Taken together, this *in vitro* PGCdMs platform has potential applications in the feasibility of performing genome-wide CRISPR screens and vaccine antigen screening.

## Materials and methods

### Chicken lines used for PGC derivation

Chicken lines providing eggs for this study were maintained at the National Avian Research Facility at the Roslin Institute under United Kingdom Home Office license under protocols approved by the local Animal Welfare and Ethics Review Board (AWERB) and carried out by United Kingdom Home Office Personal License holders under United Kingdom Home Office Project Licenses 70/8528 and PP9565661 (creation and maintenance of genetically altered chicken lines), PCD70CB48 (*Campylobacter* and *Salmonella* challenges performed at the Moredun Research Institute). The containment level was biosafety level 2 (BSL2). Fertilized Hy-line Brown layer eggs and transgenic eggs from the MAC-Green and MAC-Red lines were used to generate PGC cultures. Broiler Cobb500 eggs were obtained from Cobb-Vantress.

### PGC culture

Blood was sampled from single embryonic day 2.5 chicken eggs and cultured in FAOT medium. After 21 days in culture, PGC lines consisted of >100,000 cells and were then cryopreserved and/or used for macrophage induction experiments. Chicken germ cell culture medium contained DMEM Ca-Free high glucose (Thermo Fisher Scientific, catalog number: 21068-028), 1XB-27 supplement (Thermo Fisher Scientific, catalog number: 17504044), 1X GlutaMax (Thermo Fisher Scientific, catalog number: 35050038), 1Xnon-essential amino acids (NEAA) (Thermo Fisher Scientific, catalog number: 1114003), 1X MEM vitamin Solution (Thermo Fisher Scientific, catalog number: 11120052), 1X EmbryoMax nucleosides (Merck Millipore, catalog number: ES-008-D), 0.1mM β-Mercaptoethanol (Thermo Fisher Scientific, catalog number: 31350010), 0.2% Ovalbumin (Sigma, catalog number: A5503), 1.4mM sodium pyruvate (Thermo Fisher Scientific, catalog number: 11360039), 0.15mM calcium chloride (Merck, catalog number: C5670), and 0.01 mg/ml sodium heparin (Sigma, catalog number: H3149). The following growth factors were added to the medium just before the start of culture: human Activin A, 25 ng/ml (Peprotech, Catalog number: 120–14–10); human FGF2, 4 ng/ml (R&D Systems, Catalog number: 234-FSE-025); Ovotransferin, 10 ng/ml (Sigma, Catalog number: C7786) (Whyte et al., 2015).

### Differentiation of PGCs to macrophage-like cells

We devised three different methods of differentiating PGCs to PGCdMs (Supplementary Figure S4).

In method 1, 5 × 10^4^ PGCs were cultured in 500 µl of FAOT medium containing standard 1.8 mM CaCl_2_ in a non-fibronectin-coated 24-well plate for 48 h to form EBs. Then, 250 µl of high Ca^2+^ containing FAOT media were removed, and the loosely attached EBs were gently pipetted to dislodge from the plate surface (phase 1). These detached EBs, along with conditional media, were transferred to a fibronectin-coated 24-well plate with the addition of 250 µl hematopoietic induction media for 48 h (phase 2). Then, 250 µl hematopoietic induction media was replaced by adding 250 µl of macrophage induction media for the next 48 h. Subsequently, 250 µl of macrophage induction media was replaced with 250 ul of macrophage differentiation media, and the culturing was continued with differentiation media for approximately 30 days (phase 3).

In method 2, 5 × 10^4^ PGCs were seeded and cultured in 0.15 mM CaCl_2_ FAOT medium for 48 h on a fibronectin-coated 24-well plate. The second method did not show EBs in the culture plate like in methods 1 and 3. Cells were stimulated with 250 µl hematopoietic induction media for the next 48 h, and then 250 µl of macrophage induction media was added for the next 48 h. Subsequently, 250 µl of macrophage induction media was replaced with 250 ul of macrophage differentiation media, and the culturing was continued with differentiation media for approximately 30 days (phase 3).

In method 3, 5 × 10^4^ PGCs were seeded directly in a fibronectin-coated 24-well plate and cultured in FAOT media containing 1.8 mM CaCl_2_ for 48 h. Adherent EBs were visible. Cells were stimulated with 250 µl hematopoietic induction media for the next 48 h, and then 250 µl of macrophage induction media was added for the next 48 h. Subsequently, 250 µl of macrophage induction media was replaced with 250 ul of macrophage differentiation media, and the culturing was continued with differentiation media for approximately 30 days (phase 3).

From this point onward, the next feeding regimen was the same. Every 48 h, the established cultured cells in all three methods were re-fed with 300 µl of macrophage differentiation media and with CSF1 by removing 250 µl of the conditional macrophage differentiation media. This feeding process was continued for 2 weeks until the EBs or clustered cells were firmly attached to the substrate, and early signs of macrophage production were identified in the form of a few attached vacuolated cells or clusters of round cells with small projections.

After 14 days of culture, adherent or floating putative macrophage-like cells were visible in the supernatants. Adherent cells were isolated by pipetting and transferred to a new well. Floating macrophage-like cells were harvested, centrifuged, and transferred to a new 48-well plate in macrophage differentiation medium. For the floating cell method, parental plates were fed, and the supernatants were harvested every 72 h. Floating macrophages were typically harvested starting on day 20 and collected every 3–5 days for approximately 30–60 days. Once the floating macrophage cells fully adhered and reached 85%–90% confluence, adherent macrophages were split 1:3 every week by TrypLE (Thermo Fisher Scientific, catalog number: 12604013).

The hematopoietic induction media consists of knockout DMEM (Thermo Fisher Scientific, catalog number: 10829018) with 10% ES-grade FBS (Thermo Fisher Scientific, catalog number: 16141061), 1% chicken serum (Biosera, catalog number: CH-515), 0.2 ug/mL chicken CSF1 (Immunological Toolbox, The Roslin Institute), 25 ng/ml chicken SCF (Kingfisher Biotech, catalog number: RP1555C-005), 0.1% 100X NEAA (Thermo Fisher Scientific, catalog number: 1114003), 0.1% pyruvate (Thermo Fisher Scientific, catalog number: 11360039), 0.1% 100x Glutamax (Thermo Fisher Scientific, catalog number: 35050038), 0.5 mg/ml ovotransferrin (Sigma, catalog number: C7786), and 1% penicillin–streptomycin (Sigma, catalog number: P4458) at 37 °C with 5% CO_2_. The macrophage induction media consists of hematopoietic induction media with CSF1 and 25 ng/ml chicken IL-3 (Kingfisher Biotech, catalog number: RP1901C-005). The macrophage differentiation media consist of hematopoietic induction media with CSF1.

### Immunohistochemistry

PGCdMs were fixed in 4% (w/v) paraformaldehyde in phosphate-buffered saline (PBS) for 10 min. Fixed cells were permeabilized with PBS containing 0.3% (v/v) Triton X100 for 10 min. Fixed and permeabilized cells were blocked for 1 h at room temperature [PBS+ 0.1% (v/v) Tween (PBT) +10% serum]. Subsequently, primary antibodies mouse anti-chicken *CSF1R* (ROS-AV170, Roslin Immunological Toolbox, 1:500 dilution), rabbit anti-human caspase-3 (BD Biosciences, 1:400 dilution), and Anti LEP100 (DSHB, Antibody Registry ID: AB_2618249) (1:100 dilution) in 5% goat serum/PBT was added, and samples were incubated overnight at 4 °C. Cells were washed for 30 min in PBT and re-incubated with a secondary antibody [Goat anti-Mouse IgG (H + L), highly Cross-Adsorbed Secondary Antibody, Alexa Fluor 546, Thermo Fisher Scientific, 1:500 dilution, catalog # A-11030; Goat anti-rabbit IgG (H + L), highly Cross-Adsorbed Secondary Antibody, Alexa Fluor 546, Thermo Fisher Scientific, 1:500 dilution, catalog # A-11035) for 1 hour. Cells were washed twice for 15 min, mounted in PBS, and imaged directly. Cells were imaged using an inverted fluorescence microscope.

### Cell surface staining using flow cytometry

Adherent MAC-Green PGCdMs at 70%–85% confluency were used. Media was removed, and cells were washed with pre-warmed (37 °C) PBS to remove clumps and dead cells. PGCdMs were detached from the plate by TrypLE solution and further supplemented with FBS-containing DMEM media to neutralize TrypLE effect. Cells were washed with the FACS buffer [PBS, 2% (v/v) FBS, 5 mM EDTA] and pelleted at 300×g, 4 min, 4 °C. Cell pellets were stained with either 50 μl of diluted conjugated antibodies by IgG1 isotype control, KUL01-PE (1:1,000 dilution, Southern Biotech), KUL01-FITC (0.5 mg/ml, used at 1/100, Santa Cruz, Cat. Sc-52603), CD45-PE (0.1 mg/mL used at 1:200 dilution Southern Biotech, Cat. 8270–09), CSF1R, or MHCII-PE (1:500 or 1:100 dilution, Southern Biotech) on ice for 30 min. The cells were then pelleted at 300×g, 4 min, and 4 °C and washed twice with 75 μl FACS buffer. The final pellet was resuspended in 300 µl of FACS buffer with SYTOX Blue Nucleic Acid Stain (5 μM, Thermo Fisher Scientific #S11348 Invitrogen; 1.0 mM stock, 1/4,000 dilution) and was added immediately before flow cytometry analysis to allow for live/dead cell identification using a BD LRSFortessa flow cytometer (BD Biosciences, United Kingdom). Dead cells were excluded by SYTOX Blue staining, and doublets were discriminated against on signal processing (SSC-A/H or FSC-A/H). Data were analyzed using FlowJo v10.8 software (BD Life Sciences: Ashland, OR, United States of America).

### RNA isolation and cDNA synthesis

Total RNA was extracted from cells using a Qiagen RNeasy mini kit (#74104) according to the manufacturer’s guidelines using QIAshredder homogenizer and on-column DNase treatment. RNA concentration was measured using the NanoDrop spectrophotometer (Thermo Fisher Scientific ND-1000). cDNA was synthesized from 0.2 μg–1 μg of RNA using Promega Reverse Transcription System (catalog #A3500) at 42 °C following the manufacturer’s instructions.

### RNA extraction and RNA sequencing

Total RNA was prepared from five different PGCdMs lines, as discussed above. The Agilent RNA 6000 Pico Kit in Agilent 2100 Electrophoresis Bioanalyzer instrument and Qubit™ RNA High Sensitivity kit in Qubit 4 instrument were utilized to assess the quality and quantity of RNA. Samples displaying RNA integrity number (RIN) values between 8 and 10 were deemed appropriate for sequencing. RNA sequencing libraries were prepared from total RNA samples at the Edinburgh Genomics core facility, The University of Edinburgh. Short-read RNA-Seq libraries were generated using the Illumina Stranded Total RNA Ligation Library Prep Kit. Ribosomal RNA was depleted, and the remaining RNA molecules were subsequently purified and fragmented to a size range of 150–200 base pairs. The cleaved fragments were copied into cDNA using reverse transcriptase and random primers, as stated above. The cDNA library was then sequenced on an Illumina Novaseq SP platform to generate 2 × 100 bp paired-end reads.

### RNAseq and differential gene expression analysis

The sequencing data quality was assessed using FASTQC (version 0.11.4) (http://www.bioinformatics.babraham.ac.uk/projects/fastqc/). Sequencing adapters were then subsequently removed with Cutadapt (version 1.16) and TrimGalore (version 0.4.1) (Martin, 2011) (https://www.bioinformatics.babraham.ac.uk/projects/trim_galore/). To remove any batch effect among samples used in the analysis, we applied ComBat (version 3.36.0) for plotting only and compared PCA before and after batch adjustment (Supplementary Figure S11). As there is very little difference between the batch-adjusted values before and after, we did not use batch-corrected values for downstream data processing. The cleaned reads were aligned to the bGalGal1.mat.broiler.GRCg7b transcriptome using Salmon (version 1.10.0) (Patro et al., 2017). Using GRCg7b mitigates this risk of lacking or fragmented regions of earlier builds (e.g., GRCg6a and galGal5/4). The files Gallus_gallus.bGalGal1.mat.broiler.GRCg7b.cdna.all.fa and Gallus_gallus.bGalGal1. mat.broiler.GRCg7b.109.gtf from Ensembl were used for quantification/annotation.

The RNAseq data were available for download at GSE295795. DGE and principal component analysis were conducted in R (v4.2.3) utilizing EdgeR (v3.18) (Robinson et al., 2010). Genes with significant changes were identified, with upregulated genes defined as those showing a log2 fold change of ≥3 and an adjusted *p*-value of ≤0.05. Downregulated genes were those with a log2 fold change of ≤ −3 and an adjusted *p*-value of ≤0.05. Volcano plots were created using the ggplot2 package in R to illustrate the DEGs. Gene Ontology enrichment analysis and functional annotations were performed using DAVID (https://david.ncifcrf.gov/home.jsp). Top-ranked GO terms were displayed through plots, using the negative base 10 logarithm of *p*-values (-log10 (pval)). Sequences from Borowska et al. (accession: PRJEB56177), Idoko-Akoh et al. (accession: GSE182397), and Niu et al (accession: SRP104704) were obtained via the SRA NCBI toolkit (https://trace.ncbi.nlm.nih.gov/Traces/sra/sra.cgi?view=software), and all reads were processed as previously described (Borowska et al., 2022; Idoko-Akoh et al., 2023; Niu et al., 2017).

### Reverse transcription PCR

To detect specific transcripts, extracted RNA was reverse transcribed to cDNA as above, and then, PCR was performed using a Phusion High-Fidelity PCR Master Mix (Thermo Fisher Scientific, # F531L) for 30 cycles according to the manufacturer’s guidelines. Primers used for RT-PCR are described in Table 4.

**TABLE 4:** PCR primer list.

Gene	Forward primer (5’-3’)	Reverse primer (5’-3’)	T_m_ (°C)	Genomic DNA product size (bp)	cDNA product size (bp)
*GAPDH*	GAA​GGC​TGG​GGC​TCA​TCT​G	CAG​TTG​GTG​GTG​CAC​GAT​G	64	1,864	150
*GAPDH*	TGT​GAC​TTC​AAT​GGT​GAC​A	CAG​ATC​AGT​TTC​TAT​CAG​C	55	700	344
*NANOG*	AGC​AGA​CCT​CTC​CTT​GAC​CA	TTC​CTT​GTC​CCA​CTC​TCA​CC	64	605	186
*DAZL*	TCC​CAG​AGC​CCA​CAC​AGA​TG	AAG​TGA​TGC​GCC​CTC​CTC​TC	67	1,633	160
*TBX-T*	GAT​GAT​CAC​CAG​CCA​TTC​CT	TGC​GAG​TAT​CCA​GAC​TGC​TG	62	947	189
*TLR4*	CTA​GAA​ACC​GGG​CAG​TCA​CC	GTA​TGG​ATG​TGG​CAC​CTT​GAA​A	67.1	0	442
*CD14*	AGT​ACA​GGG​GCA​GGA​TGA​CT	AGA​GGG​ACC​TGG​GTA​GAT​GG	67.1	279	279
*MyD88*	GAG​GGA​TGA​TCC​GTA​TGG​GC	ACC​ATC​CTC​CGA​CAC​CTT​CT	67.1	0	213
*The following gene primers are from* (Sun et al., 2021)					
*MyD88*	AAG​GTG​TCG​GAG​GAT​GGT​GGT​C	GGA​ATC​AGC​CGC​TTG​AGA​CGA​G	68	2,008	120
*MHC II*	GTT​CTA​CCA​GCG​TTC​GGA​AGG​C	TCT​GAG​CGG​CGT​CCA​ACT​CC	68	101	101
*IL-10*	CAG​CAC​CAG​TCA​TCA​GCA​GAG​C	GCA​GGT​GAA​GAA​GCG​GTG​ACA​G	68	447	94
*BAX*	ACT​CTG​CTG​CTG​CTC​TCC​TCT​C	ATC​CAC​GCA​GTG​CCA​GAT​GTA​ATC	68	393	174
*TNF-a*	CAG​ATG​GGA​AGG​GAA​TGA​AC	AGA​GCA​TCA​ACG​CAA​AAG​GG	61	999	268
*IL-6*	ATG​GTG​ATA​AAT​CCC​GAT​GAA​G	CCT​CAC​GGT​CTT​CTC​CAT​AAA​C	61	1,509	153
*B-actin*	ATTGCTGCGCTCGTTGTT	CTTTTGCTCTGGGCTTCA	61	494	173
*MIF*	ATTGGCAAGATTGGAGGG	CGT​TGG​CAG​CAT​TTA​TGT​C	61	915	127
*iNOS*	AGT​GGT​ATG​CTC​TGC​CTG​CT	CCA​GTC​CCA​TTC​TTC​TTC​C	61	734	171
*MDA5*	TCA​GGA​GGA​GGA​CGA​CCA​CGA​T	TTC​CCA​CGA​CTC​TCA​ATA​ACA​G	62	2,437	168
*IL-1B*	TGGGCATCAAGGGCTACA	TCGGGTTGGTTGGTGATG	62	331	244
*Off -target site primer list*					
*CDKAL1*	TTC​CAG​TAC​CAA​GAT​GAT​GAG	CGA​TTA​TAC​AGC​CCA​GAA​ACA​C	55	426	-
*LOC423719*	TCA​TCA​AAG​CGT​AGA​AAG​GG	TGT​TTA​ATT​GCC​ACC​TCC​AC	55	453	-
*ENSGALG00010007632*	AGT​GGT​TAT​CCT​ATT​GTA​TGG​CTC	ACC​TGA​GTC​TCT​ATT​TAT​TGA​CCC	55	447	-
*ENSGALG00010027037*	TAC​CAT​CAC​CTC​CTT​TGC​TCT​G	TTC​ACA​CCA​TAA​ACA​TCC​TCC​AC	55	536	-
*GPC5*	ACA​AAG​TGA​CTT​CCT​CTG​AC	CAA​CTC​CAT​TCC​TAC​ATT​CCC	55	523	-
*CLSPN*	TTT​CTC​CAC​TGC​TGC​TTC​CC	GAA​TGA​ACT​CCA​GCC​TTG​CTC	55	403	-
*Cas9*	CAG​AGA​GCA​GGC​CGA​GAA​TA	CCG​CAT​GTT​AGC​AGA​CTT​CC	57	291	​

### LPS induction

A total of 1.5 × 10^5^ PGCdMs were plated in a single 24-well plate in macrophage differentiation medium overnight. The medium was replaced with fresh macrophage differentiation medium with or without LPS (250 ng/ml, *E. coli* 055:B5, Sigma #L2880) and incubated for 2 or 4 h. Supernatants were collected and centrifuged at 1,500 rpm for 5 min, and the upper supernatant conditioned media were filtered in a 0.22-uM filter for ELISA experiments. Stimulated cells were washed with PBS twice and detached from the ground using TrypLE solution, and the pellet was mixed thoroughly with RLT buffer (Qiagen).

### Phagocytosis assay

PGCdMs and PGCs were plated in triplicate on a non-coated 24-well plate at 2 × 10^5^ cells per well in 500 µl of macrophage maturation medium overnight. On the day of the assay, the medium was completely aspirated and replaced with 500 µl OptiMEM containing 100 μg/ml pHrodo Red Bioparticles (Thermo Fisher Scientific, #P35364). Cells were incubated at 37 °C/5% CO_2_ and imaged using a fluorescent microscope (Zeiss Axiovert 25 and 100) at various time points. Cells without beads and beads without cells or DF1 cells served as negative controls.

### Griess assay for nitric oxide production

The PGCdM and BMDM cells were reseeded at 1 × 10^6^ cells/well in triplicate wells on 6-well plates and treated with 500 ng/mL LPS *E. coli* O55:B5 (Sigma-Aldrich #L2880) for 4, 8, and 24 h, after which the supernatant was collected and stored at −20 °C until use. Nitrite (NO_2_) concentration, a stable metabolite of nitric oxide (NO) production, was measured in the Griess Reagent Kit in a 96-well flat-bottom plate following the manufacturer’s instructions (Invitrogen, United Kingdom). Briefly, 100 μl of culture supernatant from each well was transferred to a new flat-bottom 96-well plate, and an equal amount of Griess reagent was added to each well. Serial dilutions of sodium nitrite as a standard from 0.78 to 100 mM were measured in NO production to generate the standard curve. Absorbance was recorded at 570 nM using a SpectraMax 250 microplate spectrophotometer system (Molecular Devices, Sunnyvale, CA, United States of America).

### Detection of IL-10 in PGCdMs and bone-marrow-derived macrophages by capture ELISA

Chicken bone marrow cells from chicken embryonic day 18 embryos were cultured in the presence of recombinant chicken CSF1 to produce bone-marrow-derived macrophages, as described previously (Garceau et al., 2010). On day 7 of culture, 1.5 × 10^6^ BMDM cells were stimulated with 500 ng/ml of LPS in a 6-well plate to induce IL-10 secretion. Simultaneously, 1.5 × 10^6^ PGCdMs were stimulated with the LPS as described above. Cell culture supernatant from PGCdMs and BMDMs at various time points was assayed for IL-10.

A capture ELISA was performed to measure the IL-10 protein levels, as described previously (Wu et al., 2016). Briefly, assay plates (Nunc Immuno MaxiSorp, Thermo Electron LED) were coated with 3 μg/ml of capture antibody (ROV-AV164) diluted in carbonate/bicarbonate buffer (Sigma-Aldrich) and incubated overnight at 4 °C. Plates were washed and blocked with PBS containing 0.5% casein (Sigma-Aldrich) at RT for 1 h. Plates were then incubated with 50 μl of two-fold serially diluted standards (1,000 pg/ml–15.63 pg/ml) or five-fold diluted cell culture supernatant from both PGCdMs and BMDMs for 1 h, washed, and incubated with 1.0 μg/ml of biotinylated detection antibody (ROS-AV163) for 1 h. After a washing step, plates were incubated with Pierce™ Streptavidin-HRP (1:5,000, Thermo Fisher Scientific) for a further hour at RT and washed before adding substrate 1-step™ TMB (Thermo Fisher Scientific). The reaction was then stopped by a 2N sulfuric acid solution. All washing steps were performed using a microplate washer (Skan washer 400, Molecular Devices) with PBS (pH 7.2) containing 0.05% Tween 20 (PBS-T). The absorbance was read at 450 nm (550 nm as a reference to subtract the background) in a SpectraMax 250 microplate spectrophotometer system (Molecular Devices). Data were analyzed using GraphPad Prism 7.00 (GraphPad Software, Boston, Massachusetts, United States of America).

### *Salmonella* infection of PGCdMs and BMDMs

A spontaneous mutant of *Salmonella* Typhimurium strain ST4/74 resistant to nalidixic acid (*nal* ^*R*^) was used as the wild-type. An isogenic mutant deficient in the type 3 secretion system 1 (ST4/74 *nal* ^*R*^ Δ*prgH:kan*) was used to determine the extent to which uptake was due to phagocytosis stimulated by the injection of bacterial proteins regulating the actin cytoskeleton (Balic et al., 2014). Both strains were transformed with plasmid pFPV25.1 to constitutively express green fluorescent protein (GFP), which is maintained by ampicillin resistance (Valdivia and Falkow, 1996). Bacteria were routinely cultured in LB media containing antibiotics according to their resistance profile.

Infection of PGCdMs was assayed as previously described (Meek et al., 2022). Briefly, PGCdMs were plated 24 h before infection at 2 × 10^5^ per well in a 12-well tissue-culture plate in macrophage differentiation media without any antibiotics. Bacteria were grown in LB broth containing antibiotics to the stationary phase for 16 h. Bacterial counts were estimated from the optical density at a ratio of OD_600_ = 1 to 8 × 10^8^ cells/ml and confirmed retrospectively by colony-forming unit (CFU) counts. Bacteria were centrifuged at 4,000 *g* for 5 min to remove LB media and resuspended in differentiation media. The media was removed from PGCdMs and replaced with media containing the diluted bacteria at a multiplicity of infection (MOI) of 2 or untreated media as a negative control. The cells were incubated for 30 min at 37 °C/5% CO_2_.

Following two washes with PBS, the cells were then resuspended in macrophage differentiation media containing 100 μg/ml gentamicin to deplete the extracellular bacteria and further incubated for four different time points, i.e., 30 min, 3 h 30 min, 7 h 30 min, and 23 h 30 min at 37 °C/5% CO_2_. PGCdMs were washed with PBS and lysed with 1% (v/v) Triton X100. The lysates containing the intracellular bacteria were serially diluted and grown on LB agar containing 20 μg/ml nalidixic acid and 100 μg/ml ampicillin to enumerate CFU/ml.

### Staining for confocal microscopy

Round coverslips (VWR, catalog number #631-0150) were immersed in 100% ethanol to sterilize them. On the day of seeding PGCdMs, an ethanol-free dried coverslip was carefully placed in a 12-well plate, which was then coated with 0.01% poly-L-Lysine solution (R and D system, catalog number #3438-100-01) for 45 min at 37 °C/5% CO_2_. The coating solution was removed, and the well was dried in a sterile environment on the poly-L-Lysine-coated plate. Macrophages were seeded and infected with *Salmonella* infection, as described above. For staining, *Salmonella*-infected macrophages were fixed in 4% paraformaldehyde for 5 min at RT, then permeabilized in PBS/0.1% TritonX100 for 10 min before staining with Phalloidin AF647 (1:1,000) in PBS at room temperature for 30 min, and then washed for 15 min. The coverslips were washed three times with PBS and then stained with DAPI (1:10,000) before mounting with ProLong Gold antifade mountant (Molecular Probes) on glass slides and imaged on a Leica LSM10 confocal microscope.

## Confocal microscopy-based phagocytosis assay using pHrodo Red Zymosan BioParticles

WT PGCdMac, MyD88^−/−^ PGCdMac, and DF1 cells were seeded into CELLview Slide (Greiner Bio-One, Ref: 543998) at a density of 20,000 cells per well. For each cell type, two wells were prepared, representing the test and control groups.

The following day, cells in the test group were treated with pHrodo Red Zymosan BioParticles (Thermo Fisher Scientific, Ref: P35364), Hoechst 33342 (Invitrogen, Ref: H3570), and CellMask Deep Red Plasma Membrane Stain (Thermo Fisher Scientific, Ref: C10046). Briefly, cells were incubated with pHrodo beads at a final concentration of 50 μg/mL for 4 h at 37 °C. Following incubation, the culture medium was removed, and the cells were washed twice with PBS to remove non-internalized beads. Control groups were not treated with the bioparticles.

The cells in the test and control groups were then co-stained with Hoechst 33342 and CellMask Plasma Membrane Stains at a final concentration of 5 μg/mL each for 30 min. After staining, the medium was removed and the cells were washed twice with PBS. Finally, 100 μl of fresh culture medium appropriate for each cell type was added, and the cells were imaged using confocal microscopy.

### Immunofluorescence staining of macrophage markers and confocal microscopy

WT PGC-derived macrophages (PGCdMacs) and DF1 cells were seeded into 18-well μ-Slide ibiTreat (Ibidi GmbH, Ref: 81826) slides and allowed to adhere overnight. Cells were washed twice with PBS, fixed with 4% paraformaldehyde for 10 min at room temperature, and then blocked with 5% goat serum in PBS for 4 h at 4 °C to reduce non-specific antibody binding.

Cells were incubated overnight at 4 °C with primary antibodies against CSF1R (Invitrogen, Ref: MA5-28829), KUL01 (Invitrogen, Ref: MA5-28454), or LAMP1 (DHSB, Ref: 1D4B), each diluted 1:100 in 5% goat serum in PBS. After three washes with PBS, cells were incubated for 2 h at room temperature with Goat Anti-Mouse IgG (H + L), Highly Cross-Adsorbed Secondary Antibody, Alexa Fluor 546 (Thermo Fisher Scientific, Ref: A11030), diluted 1:10,000 in 5% goat serum in PBS. Nuclei were counterstained with DAPI (AAT Bioquest, Ref: 17507) at a final concentration of 5 μg/mL for 10 min. Following two washes with PBS, fresh PBS was added, and fluorescence images were acquired using a Zeiss LSM 880 confocal microscope (Carl Zeiss, Germany).

For negative controls, DF1 cells were processed in parallel with the omission of the primary antibody while all other staining conditions were maintained identically.

### Gene editing of MyD88

A pair of CRISPR/Cas9 guide sequences was designed to delete the chicken *MyD88* coding sequence. Two guide sequences targeting the translational start site and 3’UTR of the gene were identified using the CRISPOR website (http://crispor.gi.ucsc.edu/crispor.py). Oligonucleotides with overhangs were cloned into pX458-mcherry (Addgene plasmid # 161974; a gift from Joanna Wysocka) and pX458-GFP CRISPR/Cas9 vectors (eGFP is cloned to the Addgene plasmid # 62988; a gift from Feng Zhang) using methods previously described (Ran et al., 2013). To generate *MyD88* KO PGCs containing the 9.2-kb loss-of-function mutation, 100,000 PGCs were transiently transfected with 1 μg each of PX458-GFP and pX458-mcherry CRISPR/Cas9 vectors using Lipofectamine 2000 (Life Technologies). After 48 h, cells expressing both GFP and cherry were FACS-sorted into two 96-well culture plates to establish single-cell cultures. Single-cell clones were expanded and screened by PCR.

To screen for *MyD88* KO cells, single-cell clones were analyzed by PCR amplification of genomic DNA using primers (5’-CGT​CCC​AGG​TCC​GTT​TCT​G -3’ and 5’-GTG​GCT​CTG​TTG​TAA​AGT​GTT​T-3’) that bind to areas outside the deleted 9.2-kb region and amplify a PCR product of approximately 497 bp if a deletion was produced. Genotyping PCR parameters were 98 °C for 30 s, 30 cycles of 98 °C for 10 s, 62 °C for 30 s, and 72 °C for 30 s followed by a final extension of 72 °C for 5 min. PCR gel electrophoresis image data were collected using the NuGenius Gel Documentation system (Syngene). Sanger sequencing data were visualized using SeqMan Pro 17 and MegAlign Pro 1 (Lasergene 17, DNASTAR) or SnapGene version 6.2.1 (Dotmatics).

## Annexin V apoptosis assay

PGCdMs with or without MyD88 in a 24-well plate were treated with 0.125 μg/mL LPS for 1 h at 37 °C. Following treatment, cells were harvested by gentle dissociation using TrypLE (Invitrogen, catalog number: 12605010). Cells were pelleted by centrifugation at 1,500 RPM for 5 min and washed twice with cell staining buffer (BioLegend, catalog number: 420201). Then, the cells were resuspended in 100 µL of Annexin V binding buffer (BioLegend, catalog number: 422201), and apoptosis staining was performed using the PE Annexin V Apoptosis Detection dye (BioLegend, catalog number: 640934) and SYTOX Blue dead cell stain (Molecular Probes, catalog number: 34857) according to the manufacturer’s instructions, with some modifications. Briefly, 1 µL of PE-conjugated Annexin V was added to each sample. In addition to the double-stained samples, single-stained controls were prepared using Annexin V-PE alone and SYTOX Blue alone to enable fluorescence compensation during flow cytometry analysis. In addition, unstained control cells were used. Cells were gently mixed and incubated for 15 min at room temperature (approximately 25 °C) in the dark to allow Annexin V binding to exposed phosphatidylserine residues on apoptotic cells. After incubation, 400 µL of Annexin V binding buffer was added to each tube to obtain a final staining volume of 500 µL. A measure of 2 μL SYTOX Blue was added to 1,000 µL of binding buffer (2X SYTOX Blue). Five minutes before FACS analysis, 2X SYTOX Blue was added to each sample at a 1:1 ratio. Samples were analyzed by flow cytometry using a 565-nm excitation channel for Annexin V-PE and a 444-nm excitation channel for SYTOX Blue as a dead cell stain. Flow cytometry plots were analyzed using FlowJo software (FlowJo, Ashland, OR, United States of America). Control samples were used to calibrate the Fortessa X-20 instrument: unlabeled cell samples to evaluate the level of autofluorescence to adjust the instrument accordingly and cell samples individually labeled with Annexin V-PE and SYTOX Blue to define the boundaries of each cell population. Forward scatter area (FSC-A) and side scatter area or height (SSC-A) or (SSC-H) were measured.

## Data Availability

The datasets presented in this study can be found in online repositories. The names of the repository/repositories and accession number(s) can be found below: https://www.ncbi.nlm.nih.gov/geo/, GSE295795.
