# Supplementary material for: Primordial germ cell-derived macrophages as a platform to study host–pathogen interactions
Source: Front Cell Dev Biol. 2026 Sep 30;14:1684495. doi: 10.3389/fcell.2026.1684495 (PMC13661999; doi:10.3389/fcell.2026.1684495)
Supplement: Supplementary file 1 [file Supplementaryfile1.docx]

**Supplementary Information**

**Supplementary Table 1: Details of PGCdMs derivation from transgenic and wild type PGCs via three different methods**

| **Differentiation Method** | **PGC lines** | **Number of wells (n)** | **Successful PGCdM derivations (%)** | **Sex (Male +Female)** |
| --- | --- | --- | --- | --- |
| Method 1 | Transgenic | 23 | 8 (34.8) | 4M+4F |
|  | Non-transgenic | 22 | 6 (27.3) | 2F+4M |
| Method 2 | Transgenic | 31 | 11 (35.5) | 5M+6F |
|  | Non-transgenic | 37 | 6 (16.2) | 4M+2F |
| Method 3 | Transgenic | 16 | 8 (50) | 5M+3F |
|  | Non-transgenic | 16 | 9 (50.6) | 7M+2F |

**Supplementary Figure 1: Culture of GFP^+^ cells from MAC-Green PGCs using method 1**

**A.** MAC-Green PGCs formed embryoid bodies in 1.8 mM CaCl_2_ FAOT (Phase 1).

**B.** EBs adhere to and spread over a fibronectin coated surface (Phase 2).

**C.** GFP^+^ cells were visible on the well surface 4 days after EB adherence (Phase 3).

**D.** GFP^+^ cells increased in number and proliferated across the well surface (6 days after EB adherence) (Phase 3). A-D Scale bar, Scale bar, 50 µM.

n=3, data are representative of at least two independent experiments.

**Supplementary Figure 2:** Chronological development of a single green patch as a colony from an embryoid body at day 7 of adherence up to day 16. n=3, data are representative of at least two independent experiments.

**Supplementary Figure 3:** Proliferation of MAC-Green PGCdMs during initial passages on non-fibronectin-coated plates.

Pure PGCdM populations were visible at day 21 of culture and were passaged up to 7 times by day 50. n=3, data are representative of at least two independent experiments.

**Supplementary Figure 4:** Variations tested to differentiate PGCdMs from PGCs

Method 1 cultures PGCs in 1.8 mM CaCl2+ FAOT media in a non-fibronectin-coated plate for 48 hrs to make embryoid bodies. Then, embryoid bodies were transferred to fibronectin-coated plates with hematopoietic induction media for 48 hrs and then subsequently cultured in macrophage induction media for 48 hrs, and then continuously cultured in macrophage maturation media. Floating macrophages were continuously collected in the macrophage maturation media in non-fibronectin-coated plates to get adherent PGCdMs.

Method 2 cultures PGCs in normal 0.15 mM CaCl_2_ FAOT media in a fibronectin-coated plate for 48 hrs, then adding hematopoietic induction media for 48 hrs, then macrophage induction media for 48 hrs, and then continuously culturing in macrophage maturation media. Floating macrophages were continuously collected in the macrophage maturation media in non-fibronectin-coated plates to get adherent PGCdMs.

Method 3 cultures PGCs in 1.8 mM CaCl_2_ FAOT media in fibronectin-coated plate for 48 hrs to make embryoid bodies, then adding hematopoietic induction media for 48 hrs, then macrophage induction media for 48 hrs, and then continuously culturing in macrophage maturation media. Floating macrophages were continuously collected in the macrophage maturation media in non-fibronectin-coated plates to get adherent PGCdMs.

**Supplementary Figure 5.** Culture of a PGCdM from a frozen aliquot over several weeks.

One vial containing 100,000 PGCdMs was thawed and cultured until reaching 50 x 10^6^ PGCdMs.

**Supplementary Figure 6: MHC II gene expression in PGCdMs.**

**A.** The flow cytometry panel shows the gating strategy and the histogram of cultured MAC-Green PGCdMs immunostained with 1:500 and 1:100 dilutions of MHC II–PE antibody. The *CSF1R*-Tg-eGFP was on the x-axis, and the MHC II –PE was on the y-axis.

**B.** Immunocytochemistry of PGCdMs with DAPI and with or without anti-MHC II*-*PE antibody. Scale bars: 50 µM

**C.** mRNA sequencing data revealed the MHC II signature gene was upregulated > 3-fold or more relative in PGCdMs as compared to PGCs.

**Supplementary Figure 7: DF1 control cells do not express CSF1R or KUL01**

Confocal imaging of DF1 cells immunostained with anti-*CSF1R*, KUL01, and LEP100 (Lamp1) antibodies. DAPI, nuclear stain. Merge, fluorescence and brightfield. Scale bars: 10 µM.

**Supplementary Figure 8: RT-PCR analysis of PGCdMs**

cDNA from the PGCs and PGCdMs was analysed for the housekeeping gene (*GAPDH*), pluripotent marker gene (*NANOG*), germ cell marker gene (*DAZL*), and mesoderm marker (*TBXT*). Blank (-), H_2_O control.

**Supplementary Figure 9: RT-PCR expression analysis of macrophage marker genes in PGCdMs.**

cDNA from the PGCs and PGCdMs were analyzed for expression of (**A**) *MyD88, MHC II, IL10, BAX* (**B**) *TNF-a, IL6, B-actin, MIF* (**C**) *iNOS, MDA5, IL-1β*. cDNA from DF1 cells was used as a third cell line in (**C**). Blank (-), H_2_O control.

**Supplementary Figure 10:** Analysing the batch effect of samples in the PCA plots before (**A**) and after (**B**) using the ComBat (version 3.36.0).

**Supplementary Figure 11: Minus-average (MA) and Volano plots of RNA‐seq data.** MA plots were shown in between PGCdMs vs. PGC (**A**), PGCdMs vs. BMDM (**B**), and PGCdMs vs. DF1 cells (**C**). Volcano plots were shown in between PGCdMs vs. PGC (**D**), PGCdMs vs. BMDM (**E**), and PGCdMs vs. DF1 cells (**F**). In MA plots, the horizontal axis was log10 mean expression (logCPM), and the vertical axis was log2FC with a < 3-fold change (logFC). In volcano plots, the horizontal axis was logFC, and the vertical axis was FDR.

**Supplementary Figure 12: RNA expression patterns in PGCdMs**

A. The results of the Kyoto Encyclopedia of Genes and Genomes (KEGG) pathway analysis of differentially expressed genes in PGCdMs (log2Fc> 1.3-fold or more relative to PGCs) and BMDMs (BMDMs (log2Fc> 1.3-fold or more relative to PGCs)

B. Heatmap of select genes corresponding to pathways that were upregulated in PGCdMs and BMDM (log2Fc > 1.3-fold or more) relative to PGCs.

**Supplementary Figure 13: GO term pathway analysis of RNA seq data**.

The results of the Gene Ontology Terms pathway analysis showed 34 signalling pathways in PGCdMs vs PGCs (log2Fc> 4-fold) and showed 27 signalling pathways BMDMs vs PGCs (logF2c> 4-fold).

**Supplementary Figure 14:** PGCdMs were incubated with pHrodo beads and imaged for fluorescence at 0 hr, 4 hrs, 8 hrs, and 24 hrs of incubation. pHrodo beads along was used as a control. An image of the cell pellet was shown in the bottom panels. Scale bar, 100 µm.

**Supplementary Figure 15:** DF1 cells were were incubated with pHrodo beads and imaged for fluorescence at 0 hr, 4 hrs, 8 hrs, and 24 hrs of incubation. An image of the cell pellet was shown in the bottom panels. Scale bar, 100 µm.

**Supplementary Figure 16:** Sanger sequencing of a *MyD88^-/-^* colony with forward and reverse sequencing primers to determine the deleted region. Sanger sequencing shown for clone #1 indicates a deletion of the translational reading frame.

.

**Supplementary Figure 17: RT-PCR expression analysis of TLR4 and MyD88 in PGCdMs in MyD88***^-/-^* **PGCdMs.** cDNA from the PGCdMs with or without MyD88 was analyzed for expression of (**A**) GAPDH, (**B**) TLR4, (**C**) MyD88. NTC, H2O control as blank. RT+, reverse transcriptase. RT-, no reverse transcriptase control. The amplified GAPDH PCR product is 344 bp in cDNA and 700 bp in genomic DNA. The amplified PCR product in TLR4 is 442 bp, and MyD88 is 213 bp.

**Supplementary Figure 18: Investigating off-target activity of MyD88 sgRNA1.**

**A**. Sequence alignment of putative off-target sites of MyD88 sgRNA1.

**B**. Genotyping PCR of the putative off-target sites of MyD88 sgRNA1 and sgRNA2.

**C.** Sequencing traces of three off-target sites, CDKAL1, LOC423719, and ENSGALG00010007632 (short code 10007632).

**Supplementary Figure 19:** **Screening the integration of plasmid backbones in MyD88 ^-/-^ PGCdMs.**

**A**. Primers chosen for Cas9 plasmid backbone PCR amplification.

**B**. GAPDH housekeeping gene amplification in pX458 plasmid, MyD88 ^+/+^ and MyD88 ^-/-^PGCdMs.

**C**. Cas9 amplification in in pX458 plasmid, MyD88 ^+/+^ and MyD88 ^-/-^PGCdMs. NTC, H20 control.

**Supplementary Figure 20:** **Investigating the presence of MyD88 transcript in PGCdMs.**

A. cDNA sequence of MyD88 transcript.

B. Sequencing trace of MyD88 transcript.

**Supplementary Figure 21:** PGCdMs and DF1 cells were incubated with pHrodo beads and imaged for fluorescence 4 hrs. Z-sections indicates that the pHrodo beads are contained within the PGCdMs.. Scale bar, 10 µm.

**Supplementary Figure 22: Annexin V apoptosis assay in PGCdMs with or without MyD88, stimulated with or without LPS (Only taking intact cells)**

**A**. Single cells were gated from the intact cells population and further assessed according to their differential staining of Annexin V-PE and SYTOX Blue. Live cells were identified as being negative for both Annexin V-PE and SYTOX Blue, early apoptotic cells as positive for Annexin V-PE only, late apoptotic cells as positive for both Annexin V-PE and SYTOX Blue, and dead/necrotic cells as positive for SYTOX Blue only.

**B**. Percentage of live, early apoptotic, late apoptotic, and dead/necrotic PGCdMs cells dually stained with Annexin V-PE and the SYTOX Blue dead cell stain before and after treatment with 1 *μ*M LPS for 1 hour.

**C**. As in B, but for MyD88 KO PGCdMs cells

**D**. Showing % live cells in control and KO PGCdMs after adding LPS in the Annexin V assay. n = 2.

**E**. Showing % of early apoptotic cells in control and KO PGCdMs after adding LPS in the Annexin V assay. n = 2.

**F**. Showing % of late apoptotic cells in control and KO PGCdMs after adding LPS in the Annexin V assay, n = 2.

**Supplementary Figure 23 Annexin V apoptosis assay in PGCdMs with or without MyD88, stimulated with or without LPS (Taking total cells, excluding debris)**

**A.** Single cells were gated from the all visible cells population and further assessed according to their differential staining of Annexin V-PE and SYTOX Blue. Live cells were identified as being negative for both Annexin V-PE and SYTOX Blue, early apoptotic cells as positive for Annexin V-PE only, late apoptotic cells as positive for both Annexin V-PE and SYTOX Blue, and dead/necrotic cells as positive for SYTOX Blue only.

**B**. Percentage of live, early apoptotic, late apoptotic, and dead/necrotic PGCdMs  dually stained with Annexin V-PE and the SYTOX Blue dead cell stain before and after treatment with 1 *μ*M LPS for 1 hour. (**C**) As in B, but for MyD88 KO PGCdMs  .

**D**. Showing % live cells in control and KO PGCdMs after adding LPS in the Annexin V assay. n = 2.

**E**. Showing % of early apoptotic cells in control and KO PGCdMs after adding LPS in the Annexin V assay. n = 2.

**F**. Showing % of late apoptotic cells in control and KO PGCdMs after adding LPS in the Annexin V assay. n = 2.

**G**. Showing % of dead/necrotic cells in control and KO PGCdMs after adding LPS in the Annexin V assay. n = 2.
